# Mechanism and Selectivity of Electrochemical Reduction of CO_2_ on Metalloporphyrin Catalysts from DFT Studies

**DOI:** 10.3390/molecules28010375

**Published:** 2023-01-02

**Authors:** Zaheer Masood, Qingfeng Ge

**Affiliations:** School of Chemical and Biomolecular Sciences, Southern Illinois University, Carbondale, IL 62901, USA

**Keywords:** metalloporphyrin complexes, electrochemical CO_2_ reduction, HCOO^−^ selectivity, CO selectivity, HER, ergoneutral pH

## Abstract

Electrochemical reduction of CO_2_ to value-added chemicals has been hindered by poor product selectivity and competition from hydrogen evolution reactions. This study aims to unravel the origin of the product selectivity and competitive hydrogen evolution reaction on [MP]^0^ catalysts (M = Fe, Co, Rh and Ir; P is porphyrin ligand) by analyzing the mechanism of CO_2_ reduction and H_2_ formation based on the results of density functional theory calculations. Reduction of CO_2_ to CO and HCOO^−^ proceeds via the formation of carboxylate adduct ([MP-COOH]^0^ and ([MP-COOH]^−^) and metal-hydride [MP-H]^−^, respectively. Competing proton reduction to gaseous hydrogen shares the [MP-H]^−^ intermediate. Our results show that the pK_a_ of [MP-H]^0^ can be used as an indicator of the CO or HCOO^−^/H_2_ preference. Furthermore, an ergoneutral pH has been determined and used to determine the minimum pH at which selective CO_2_ reduction to HCOO^−^ becomes favorable over the H_2_ production. These analyses allow us to understand the product selectivity of CO_2_ reduction on [FeP]^0^, [CoP]^0^, [RhP]^0^ and [IrP]^0^; [FeP]^0^ and [CoP]^0^ are selective for CO whereas [RhP]^0^ and [IrP]^0^ are selective for HCOO^−^ while suppressing H_2_ formation. These descriptors should be applicable to other catalysts in an aqueous medium.

## 1. Introduction

Carbon dioxide emissions from fossil fuel combustion have resulted in an accelerated increase in atmospheric CO_2_ levels, and consequently, serious climate impacts and ocean acidification [1,2]. Conversion of CO_2_ to value-added chemicals and fuels with sustainable and renewable energy sources helps to mitigate increasing CO_2_ levels in the atmosphere [3,4,5]. Among the options for CO_2_ conversion and utilization, electrochemical reduction driven by electricity generated from renewable energy sources such as sunlight and wind is a promising approach [6,7,8]. However, poor product selectivity and competing hydrogen evolution reactions (HER) due to proton reduction are among the major obstacles to the deployment of electrochemical reduction of CO_2_ [9,10,11].

Tuning of the active site of a molecular catalyst and optimization of the operating pH have been used to control selectivity while suppressing HER. The intrinsic activity of a molecular catalyst determines the reduction potential of the electron transfer steps, pK_a_ of protonated intermediates, and the binding energy of the adsorbate. This activity can be tuned by changing the metal ion or modifying the ligands. Computational electrocatalysis can provide a mechanistic understanding of the reaction chemistry, predict the reduction potential of a particular catalyst, and determine the pK_a_ value and adsorption strength of intermediates. This information will in turn help screening of the metal centers and ligands and guide the selection of experimental conditions, such as applied potential and pH.

Molecular complexes, as homogenous catalysts, can reduce CO_2_ to CO or HCOO^−^ and proton to gaseous hydrogen (two electron-reduction products). CO can be fed to the Fischer–Tropsch process [12] for the synthesis of alkanes, whereas HCOO^−^ can be protonated to formic acid and used as a commodity chemical or fuel in a direct formic acid fuel cell [13]. Electrocatalysts such as pincer complexes [14,15,16,17,18], cyclam complexes [19,20,21,22], porphyrins [23,24,25,26,27], phthalocyanines and iron carbonyl [28,29,30,31,32] as well as metal–organic frameworks (MOFs) [33,34] have been tested for CO_2_ reduction.

Electrochemical reduction of CO_2_ to CO/HCOO^−^ and the competing H^+^ reduction to H_2_ on metal complexes (([ML]^n^), M = metal, L = ligand) starts from the one-electron reduction of [ML]^n^ to form [ML]^(n−1)^ [35,36,37]. This is followed by either CO_2_ binding to [ML]^(n−1)^ to form [ML-COO]^(n−1)^, leading toward CO cycles, or proton binding to [ML]^(n−1)^ to form ([ML-H]^n^). This results in a HCOO^−^/H_2_ cycle. The subsequent one-electron and two-proton transfer to [ML-COO]^(n−1)^ lead to the formation of metal carbonyl [LM-CO]^0^. Desorption of CO from [LM-CO]^0^ completes the CO cycle. On the other hand, one-electron reduction of [ML-H]^(n−1)^ leads to metal hydride ([ML-H]^(n−1)^), which then reacts with CO_2_ or H^+^ to form HCOO^−^ or H_2_ [14,20,38,39,40]. The intrinsic property of the metal complex as well as the operating pH and applied potential determines which pathway the reaction will follow and what product will form from the electrochemical reduction of CO_2_.

Previous studies show that the strength of a Brönsted acid is very important in the selective reduction of CO_2_ to CO and suppression of proton reduction in nonaqueous solvents [41,42]. On the other hand, an aqueous medium is essential to keep the practical deployment of electrochemical CO_2_ reduction green, as the use of an organic solvent may have an unintended impact on the environment. However, the abundance of protons in an aqueous medium would cause proton reduction to compete with CO_2_ reduction, resulting in reduced faradaic efficiency for CO_2_ reduction.

On the Fe porphyrin catalyst, both H_2_ and CO can be produced in an aqueous solution, and their relative productivity depends on the pH and nature of the buffer [43]. On Co porphyrin, hydrogen was produced at pH < 3.0, whereas CO dominated at higher pH [44]. On Rh porphyrin, HCOO^−^ is the main product at pH = 6.8, whereas a significant amount of hydrogen was formed at pH < 4 [27]. These studies show that both the metal center of the catalyst and pH play roles in determining product selectivity.

Herein, we mapped out the pathways of CO_2_ reduction to CO and HCOO^−^, along with the proton reduction to H_2_ on [MP]^0^ catalysts (M = Fe, Co, Rh, and Ir; P is the porphyrin ligand) based on the results of density functional theory (DFT) calculations. The results indicate that selective reduction of CO_2_ to CO or HCOO^−^ while suppressing HER in an aqueous medium can be achieved by selecting the metal center in the porphyrin complexes and optimizing the operating pH. Furthermore, the ergoneutral pH can be used to determine the threshold pH above which hydrogen evolution becomes unfavorable.

## 2. Results

**Scheme 1:**
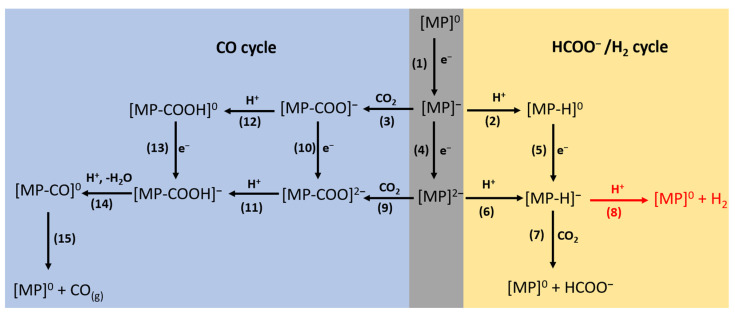
Schematic reaction steps of the electrocatalytic reduction of CO_2_ to CO (blue background) and HCOO^−^ (yellow background). The yellow regions also contain proton reduction to hydrogen (red part). The grey region shows common intermediates shared by both cycles. M = Fe, Co, Rh and Ir; P = porphyrin ligand.

Electrochemical reduction of CO_2_ to CO and HCOO^−^, including the competing hydrogen evolution reaction, was studied using the reaction mechanism shown in Scheme 1. In Scheme 1, the catalytic cycle starts from the reduction of the catalyst ([MP]^0^) to the reduced form ([MP]^−^) at the corresponding reduction potential (E°) (Step 1) [35,36,37]. Next, the pathway branches into three possibilities: (i) a proton reacts with [MP]^−^ to form a proton adduct ([MP-H]^0^) (Step 2), (ii) CO_2_ binds [MP]^−^ to form a CO_2_ adduct ([MP-COO]^−^) (Step 3) and (iii) further reduction of [MP]^−^ to [MP]^2−^ (Step 4). Proton transfer to [MP]^2−^ (Step 6) leads to the formation of metal hydride ([MP-H]^−^), whereas CO_2_ binding to [MP]^2−^ leads to [MP-COO]^2−^ (Step 9). [MP-H]^−^ can also be formed by the reduction of [MP-H]^0^ (Step 5). Further, [MP-H]^−^ can react with either CO_2_ to form HCOO^−^ (Step 7) or H^+^ to produce hydrogen (Step 8). Completion of Steps 7 and 8 regenerates the catalyst in the form of [MP]^0^.

As shown in Scheme 1, [MP]^−^ and [MP]^2−^ are the common intermediates for both the CO and HCOO^−^/H_2_ cycles. The CO_2_ binding on the metal center of these complexes (Steps 3 and 9) starts the CO cycle. These may be followed by one-electron reduction ([MPCOO]^−^ + e^−^, Step 10) and protonation ([MP-COO]^2−^ + H^+^, Step 11), protonation ([MPCOO]^−^ + H^+^, Step 12) and one-electron reduction ([MPCOOH]^0^ + e^−^, Step 13). These pathways converge at the formation of [MP-COOH]^−^. Protonation of [MPCOOH]^−^ cleaves the C–O bond, resulting in [MP-CO]^0^ and H_2_O (Step 14). Desorption of CO from [MP-CO]^0^ regenerates the catalyst and completes the CO cycle (Step 15).

The first reduction potentials (step 1, E°, V) and second reduction potentials (Step 4, V) in Scheme 1 for [FeP]^0^, [CoP]^0^, [RhP]^0^, and [RhP]^0^ are presented in Table 1. Clearly, the first reduction potentials for [FeP]^0^ and [CoP]^0^ are below −1 V and significantly more negative than those of [RhP]^0^ and [IrP]^0^. Those negative potentials at −1.24 V and −1.28 V make [FeP]^0^ and [CoP]^0^ active for CO_2_ reduction. The calculated E° for [FeP]^0^/[FeP]^−^ and [CoP]^0^/[CoP]^−^ are in close agreement with the corresponding experimental values of −1.3 V [27] and −1.10 V [45], respectively. A DFT-calculated E° = −1.15 V for [CoP]^0^/[CoP]^−^ has been reported [40]. Moreover, the second reduction potentials are more negative than the corresponding first reduction potentials, indicating that the formation of [MP]^2−^ is less favorable and Step 4 is unlikely to happen under a typically applied potential of CO_2_ reduction (−1 V to −1.5 V) [46,47]. Consequently, [MP]^2−^ is less likely to have a significant contribution to either the CO or HCOO^−^/H_2_ cycle.

The subsequent steps (Scheme 1) involve either protonation of [MP]^−^ and [MP]^2−^ (Step 2 and 6) or CO_2_ binding on [MP]^−^ and [MP]^2−^ (Step 3 and 9), branching the reaction to the HCOO^−^/H_2_ and CO cycles, respectively. As shown in Table 1, the second reduction potentials of [MP]^−^ (Step 4) are more negative than the corresponding first reduction potentials, indicating that protonation of [MP]^2−^ (Step 6) and CO_2_ binding on [MP]^2−^ (Step 9) are unlikely to contribute to the overall reaction in either of the cycles. Consequently, Steps 2 and 3 will be the dominant pathways. If protonation of [MP]^−^ is favorable, the reaction will proceed toward the HCOO^−^/H_2_ cycle via [MP-H]^0^ formations. Otherwise, CO_2_ will be activated upon binding on [MP]^−^, steering the reaction toward the CO cycle. Therefore, the reaction free energy of protonation of [MP]^−^ to [MP-H]^0^ (Step 2) will dictate the selectivity between the CO and HCOO^−^/H_2_ cycles.

The proton transfer step (Step 2) is represented by Equation (1). The dependence of the reaction free energy, ΔG°_rxn_(PT), on the pH and pK_a_ of [MP-H]^0^ can be derived with Equation (2). Equation (2) clearly shows that ΔG°_rxn_(PT) can be regulated by adjusting pH relative to the pK_a_ of [MP-H]^0^. Consequently, the pK_a_ of [MP-H]^0^ can be used as a descriptor for HCOO^−^/H_2_ selectivity. Since ΔG°_rxn_(PT) is positive for pH > pK_a_ of [MP-H]^0^, proton binding to the metal center will not be spontaneous, making [MP]^−^ available for CO_2_ binding. For pH < pK_a_ of [MP-H]^0^, ΔG°_rxn_(PT) will become negative, indicating the spontaneity of protonation and initiation of the HCOO^−^/H_2_ cycle.

[MP]^−^ + H^+^ → [MP-H]^0^: ΔG°_rxn_(PT) (1)

ΔG°_rxn_(PT) = 2.303RT(pH − pK_a_[MP-H]^0^) (2)

### 2.1. HCOO^−^/H_2_ Cycle

The calculated pK_a_ values of [MP-H]^0^ and [MP-H]^−^ for all catalysts are presented in Table 2. The pK_a_ values of [FeP-H]^0^ and [CoP-H]^0^ are 2.45 and 6.29. At pHs lower than 2.45 and 6.29 for [FeP]^0^ and [CoP]^0^ catalysts, respectively, the formation of [FeP-H]^0^ and [CoP-H]^0^ becomes possible and leads to the HCOO^−^/H_2_ cycle. One-electron reduction of [FeP-H]^0^ and [CoP-H]^0^ forms [FeP-H]^−^ and [CoP-H]^−^ at reduction potentials of −0.74 V and −1.12 V, respectively. These reduction potentials are less negative than the corresponding E°, indicating the spontaneity of these steps. The calculated pK_a_ values of [FeP-H]^−^ and [CoP-H]^−^ are 19.45 and 13.45, indicating that [FeP-H]^−^ and [CoP-H]^−^ are stable in the aqueous medium and will not deprotonate. Under the basic condition, hydride transfer from [MP-H]^−^ to CO_2_ or H_2_O results in HCOO^−^ or H_2_, respectively (Steps 7 and 8, Equations (3) and (4)) [14,39].

[MP-H]^−^ + CO_2_ → [MP]^0^ + HCOO^−^: ΔG°_rxn_(HCOO^−^) (3)

[MP-H]^−^ + H_2_O → [MP]^0^ + OH^−^ + H_2_: ΔG°_rxn_(H_2_)_(H2O)_ (4)

**Figure 1:**
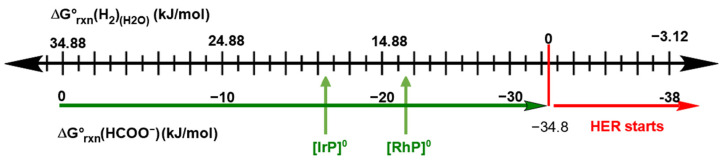
Graphical representation of the relationship of ΔG°_rxn_(HCOO^−^) and ΔG°_rxn_(H_2_)_(H2O)_.

There is a constant difference of 34.88 kJ/mol between ΔG°_rxn_(HCOO^−^) and ΔG°_rxn_(H_2_)_(H2O)_, ΔG°_rxn_(H_2_)_(H2O)_ = ΔG°_rxn_(HCOO^−^) + 34.88 kJ/mol [20], as presented graphically in Figure 1. This result indicates that HCOO^−^ formation is always more thermodynamically favorable than H_2_ formation. On [FeP-H]^−^ and [CoP-H]^−^, ΔG°_rxn_(HCOO^−^) are −138.50 kJ/mol and −158.42 kJ/mol, respectively, whereas ΔG°_rxn_(H_2_)_(H2O)_ are −103.62 kJ/mol and −123.52 kJ/mol, respectively. These values indicate that the reactions for both HCOO^−^ and H_2_ formation are spontaneous. Indeed, hydrogen production on [FeP]^0^ and [CoP]^0^ has been observed at pH = 3 experimentally [27,44].

The calculated pK_a_ values of [RhP-H]^0^ and [IrP-H]^0^ are 10.07 and 11.25 (Table 2), respectively, indicating that a pH below those values will make HCOO^−^/H_2_ thermodynamically favorable. The reduction potentials for [RhP-H]^0^ and [IrP-H]^0^ to [RhP-H]^−^ and [IrP-H]^−^ are −1.19 V and −1.26 V, respectively, and the pK_a_ values of [RhP-H]^−^ and [IrP-H]^−^ are 17.53 and 23.75, respectively, indicating that the resulting [RhP-H]^−^ and [IrP-H]^−^ will not deprotonate. The calculated ΔG°_rxn_(HCOO^−^) for the [RhP]^0^, and [IrP]^0^ catalysts are −21.30 kJ/mol and −16.51 kJ/mol, respectively. The negative reaction free energies indicate that transferring a hydride from [MP-H]^−^ to CO_2_ to form HCOO^−^ is favorable on the [RhP]^0^ and [IrP]^0^ catalysts.

The calculated ΔG°_rxn_(H_2_)_(H2O)_ are 13.58 kJ/mol and 18.37 kJ/mol for [RhP-H]^−^ and [IrP-H]^−^, respectively. The positive ΔG°_rxn_(H_2_) for [RhP]^0^ and [IrP]^0^ catalysts indicate that hydrogen evolution is unfavorable. The negative values of ΔG°_rxn_(HCOO^−^) and positive values of ΔG°_rxn_(H_2_)_(H2O)_ show that [RhP]^0^ and [IrP]^0^ are selective to HCOO^−^ while suppressing HER, as shown in Figure 1.

The reaction free energy of [MP-H]^−^ with a proton to produce H_2_ (Step 8, Scheme 1, Equation (5)) depends on the pH.

[MP-H]^−^ + H^+^ → [MP]^0^ + H_2_: ΔG_rxn_(H_2_) (5)

The pH at which ΔG_rxn_(H_2_) is zero is referred to as ergoneutral pH. A quantitative relationship between ΔG°_rxn_(HCOO^−^) and the ergoneutral pH has been derived:

pH(ergoneutral) = (41.84 kJ/mol − ΔG°rxn(HCOO^−^))/5.70 kJ/mol (6)

**Figure 2:**
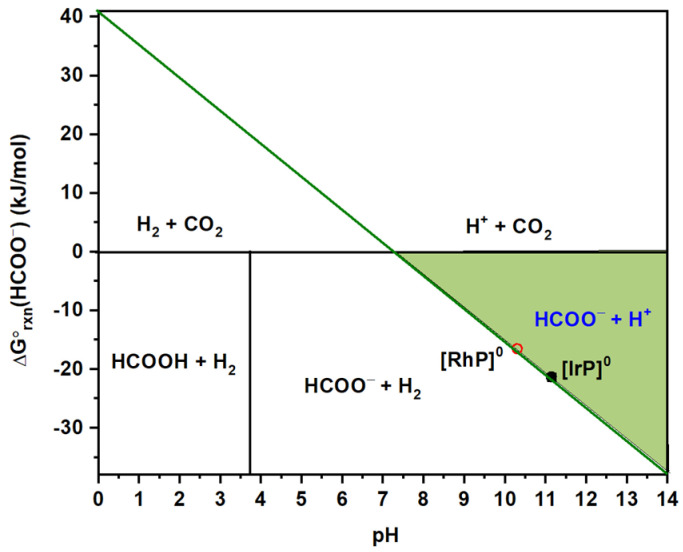
A trend of ΔG°_rxn_(HCOO^−^) and ergoneutral pH shows the distribution of CO_2_ and H^+^ reduction products.

Equation (6) determines the pH at which ΔG_rxn_(H_2_) becomes zero. At a pH less than the ergoneutral pH, hydrogen evolution prevails. Equation (6) was used to construct the product distribution diagram shown in Figure 2. The vertical line at pH = 3.75 separates the HCOOH and HCOO^−^ zone and the horizontal line at ΔG°_rxn_(HCOO^−^) = 0 divides the zone of gaseous CO_2_ and HCOOH/HCOO^−^. The green diagonal line divides the zone between H_2_ and H^+^. The green region in Figure 2 corresponds to the selective reduction of CO_2_ to HCOO^−^ where HER is suppressed. Similar schemes have been used to determine the optimum pK_a_ value of the acids for non-aqueous solvents [48,49].

The calculated ergoneutral pHs for [FeP-H]^−^, [CoP-H]^−^, [RhP-H]^−^ and [IrP-H]^−^ from Equation (6) are 31.69, 35.19, 11.14 and 10.30, respectively. These values show that HCOO^−^ and gaseous hydrogen will be produced on [FeP-H]^−^ and [CoP-H]^−^, as the calculated ergoneutral pHs are 31.69 and 35.19, respectively (>>14). In contrast, the [RhP-H]^−^ and [IrP-H]^−^ catalysts will produce HCOO^−^ selectively at a very basic pH. Proton reduction becomes possible at pH < 11.14 and 10.30 on [RhP-H]^−^ and [IrP-H]^−^, respectively. The calculated ergoneutral pHs have been incorporated in Figure 2 for [RhP-H]^−^ and [IrP-H]^−^. These predictions are consistent with the experimental observation that HER was dominant on [RhP]^0^ at pH < 6 [27]. In summary, these results show that [RhP]^0^ and [IrP]^0^ are selective to CO_2_ reduction to HCOO^−^ in a basic pH and active for HER at an acidic pH. The ergoneutral pH values for [FeP-H]^−^ and [CoP-H]^−^ are outside the limits of Figure 2 and were not plotted.

### 2.2. CO Cycle

The pK_a_ values of [MP-H]^0^ determine the lowest pH at which protonation of the metal center of the complexes is not spontaneous, freeing the metal center for CO_2_ binding. These values are 2.45, 6.28, 10.07 and 11.15 for [FeP-H]^0^, [CoP-H]^0^, [RhP-H]^0^ and [IrP-H]^0^, respectively. These pK_a_ values indicate that the reaction can be steered to the CO cycle on [FeP]^0^ and [CoP]^0^ at pHs higher than 2.45 and 6.28, respectively. On the other hand, we will not examine the details of the CO cycle on [RhP]^0^ and [IrP]^0^ as the pHs of 10.07 and 11.15 are too high. Instead, we will focus our discussion of the CO cycle on the [FeP]^0^ and [CoP]^0^ catalysts.

**Figure 3:**
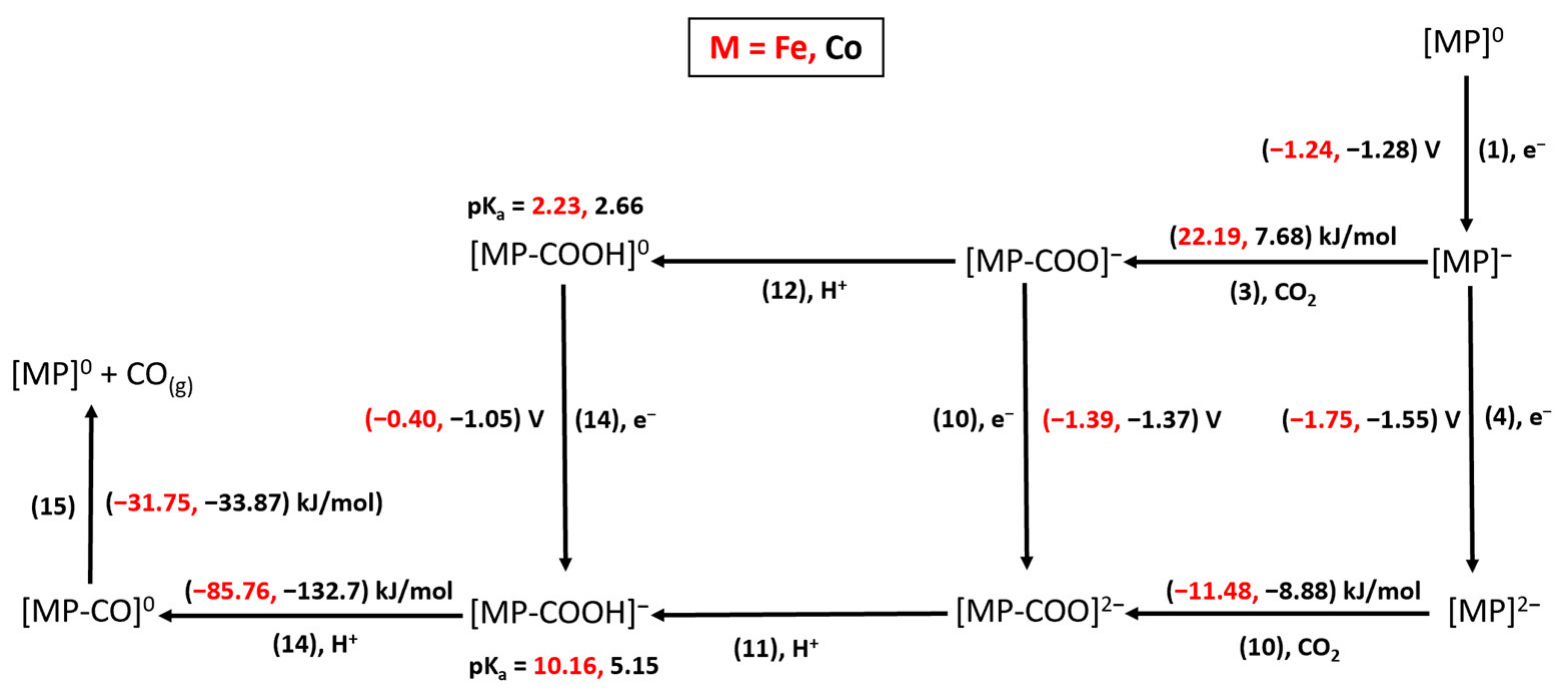
Electrochemical reduction of CO_2_ to CO on [FeP]^0^ and [CoP]^0^. Red parameters correspond to the CO cycle on [FeP]^0^ and black correspond to [CoP]^0^.

The CO cycle is replotted in Figure 3, with the numbers in red corresponding to steps on [FeP]^0^. The cycle on [FeP]^0^ starts with the reduction of [FeP]^0^ to [FeP]^−^ at −1.24 V. The formation of [FeP-COOH]^−^ can happen through a number of electron transfer steps in different orders combined with CO_2_ binding and proton transfer. The reduction of [FeP]^−^ requires a significantly more negative potential (−1.75 V) and is unlikely to play a role in CO formation. Starting from [FeP]^−^, the free energy of binding of CO_2_ on [FeP]^−^ is 22.19 kJ/mol. The pK_a_ value of [FeP-COOH]^0^ is 2.23, indicating that protonation of [FeP-COO]^−^ becomes spontaneous only at a pH < 2.23. Further reduction of [FeP-COOH]^0^ to [FeP-COOH]^−^ happens at a potential of −0.40 V. As we showed above, a low pH of 2.23 will make the HCOO^−^/H_2_ pathway competitive and, therefore, CO formation through this reduction less likely for CO formation. Generally, electrochemical CO_2_ reduction happens in a slightly acidic or neutral pH. At this pH, the pathway following [FeP]^0^ → [FeP]^−^ → [FeP-COO]^−^ + H^+^ → [FeP-COOH]^0^ + e^−^ → [FeP-COOH]^−^ is likely to be the favorable pathway. Further protonation of [FeP-COOH]^−^ leads to [FeP-CO]^0^ and H_2_O (Step 14). This step is exergonic, with a reaction free energy of −85.76 kJ/mol. CO desorption from [FeP-CO]^0^ is also exergonic with a desorption free energy of −31.75 kJ/mol (Step 15). Therefore, CO_2_ reduction to CO can happen at −1.24 V in a slightly acidic or neutral pH on [FeP]^0^. This conclusion is consistent with the experimental results reported by Costentin et al. [37].

The CO cycle on [CoP]^0^ follows a similar sequence of steps. Since the reduction of [CoP]^−^ requires a more negative potential (−1.55 V), CO_2_ activation will not likely proceed via [CoP]^2−^ at an applied potential of E° (−1.28). Instead, the CO cycle will primarily occur through CO_2_ activation on [CoP]^−^ (Step 3). The binding free energy of CO_2_ on [CoP]^−^ to form [CoP-COO]^−^ is 7.68 kJ/mol. The pK_a_ of [CoP-COOH]^0^ is 2.66, indicating that protonation of [CoP-COO]^−^ to form [CoP-COOH]^0^ requires a pH < 2.66. This pK_a_ value is in close agreement with the value (3 ± 0.4) reported by Göttle and Koper [50]. Further reduction of [CoP-COOH]^0^ to [CoP-COOH]^−^ occurs at a potential of −1.05 V. This alternate proton and electron transfer pathway requires a very low pH at which protonation of [CoPCOO]^−^ becomes spontaneous. The alternative sequential electron–proton transfer pathway, i.e., [CoP-COO]^−^ → [CoP-COO]^2−^ → [CoP-COOH]^−^, requires a potential of −1.37 V (Step 10), which is more negative than the first reduction potential (Step 1, −1.28 V). At a neutral or slightly alkaline condition, the proton and electron transfer process, i.e., [CoP-COO]^−^ → [CoP-COOH]^0^ → [CoP-COOH]^−^, although not spontaneous, would be the most favorable pathway. The reaction of [CoP-COOH]^−^ with a proton to form [CoP-CO]^0^ and H_2_O (Step 14) is spontaneous with a reaction-free energy of −132.7 kJ/mol. Desorption of CO is also spontaneous (Step 15). These results indicate that CO formation from electrochemical CO_2_ reduction at an applied potential of −1.28 V occurs via a series of electron and proton transfer steps at neutral pH or slightly alkaline conditions.

## 3. Discussion

The initial competitive binding of proton and CO_2_ to [MP]^−^ determines the pathway that the reaction will follow. Protonation will lead to the HCOO^−^/H_2_ cycle. A more nucleophilic metal center tends to bind a small proton more preferably than CO_2_, making the reaction follow the HCOO^−^/H_2_ cycle. On the other hand, a less nucleophilic metal center will bind CO_2_ and steer the reaction toward the CO cycle. The charge distribution and electrostatic potential on the metal center in the complex provide an understanding of the preference for either proton or CO_2_ binding [51]. The results of Bader charge analysis for [MP]^0^ and [MP]^−^ in Table 3 show that the electron density is significantly delocalized on the porphyrin ligand in [FeP]^−^ and [CoP]^−^ following the one-electron reduction. In contrast, the electron density is shared between the metal center and ligands in [RhP]^−^ and [IrP]^−^ following one-electron reduction from [RhP]^0^ and [IrP]^0^, with the electron density on the metal center being slightly larger. The increased electron density at the metal center (Rh and Ir) increases their nucleophilicity, making them more attractive to protons and favorable for [MP-H]^0^ formations. The low electron density at the metal center of [FeP]^−^ and [CoP]^−^ following the reduction does not enhance their reactivity toward protons, leaving them open to CO_2_ adsorption and its subsequent reduction to CO.

The electrostatic potentials of [MP]^−^ in Figure S1 (Supplementary Materials) show that the negative electrostatic potential is centered on Rh and Ir in [RhP]^−^ and [IrP]^−^, making these metal centers prone to proton binding. In contrast, the negative electrostatic potential is delocalized among the metal centers and the porphyrin ligands in [FeP]^−^ and [CoP]^−^, reducing their affinity toward protons. 

**Figure 4:**
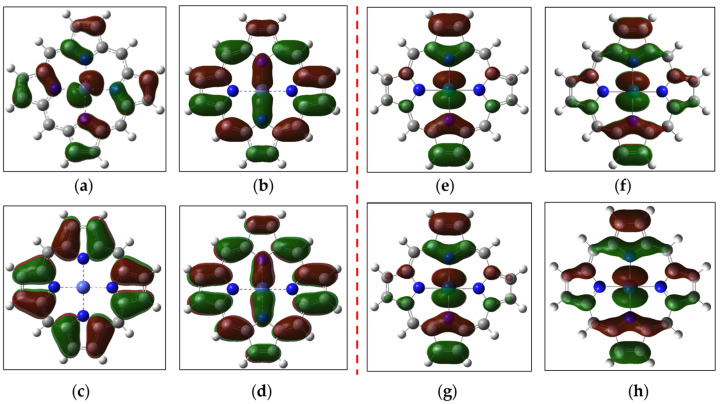
The HOMOs of (**a**) [FeP]^0^, (**b**) [FeP]^−^, (**c**) [CoP]^0^, (**d**) [CoP]^−^, (**e**) [RhP]^0^, (**f**) [RhP]^−^, (**g**) [IrP]^0^ and (**h**) [IrP]^−^. (Isosurface value = 0.02).

In order to understand the contribution of the frontier orbitals to the reactivity, we plotted the highest occupied molecular orbitals of [MP]^0^ and [MP]^−^ in Figure 4. The HOMOs of [FeP]^−^ and [CoP]^−^ show that there is a significant overlap between the p_z_ orbitals of the two nitrogen atoms of the porphyrin and d_xz_ orbitals of the metal center. The overlap between d_xz_ and p_z_ orbitals was facilitated by the two short (N–M) bonds (~1.980 Å) in [FeP]^−^ and [CoP]^−^. The other two (N–M) bonds are longer, with bond lengths of 2.032 Å and 2.018 Å in [FeP]^−^ and [CoP]^−^, respectively. There is a lack of such overlap in the HOMOs of [RhP]^−^ and [IrP]^−^. The longer N–M bonds (2.041 Å) prevented significant overlap in [RhP]^−^ and [IrP]^−^. The orbital overlap between the metal center and its ligands provides a channel for the electrons to be shared and will affect the redox activity of the transition metal–porphyrin complex. Lack of such orbital overlap will hinder the electron channeling between the metal center and ligands and results in the increased nucleophilicity of the metal centers, steering the reaction towards the HCOO^−^/H_2_ cycle.

The first reduction potentials of the metal complex also offer some insights into the preference for proton/CO_2_ binding. The first reduction potentials of [CoP]^0^ and [FeP]^0^ are −1.24 V and −1.28 V, respectively, whereas those of [RhP]^0^ and [IrP]^0^ are only −0.02 V and −0.26 V, respectively. It has been shown that CO_2_ binding is favored at a potential more negative than −1 V [35,52]. The more negative reduction potentials for [CoP]^0^ and [FeP]^0^ can be attributed to the more effective charge delocalization between the metal center and ligands. Further reduction of the activated CO_2_ is driven by the applied potential for the electron transfer steps. In contrast, the proton transfer steps are influenced by the chemical potential of the protons.

In summary, the electronic structure analyses shed additional light on the mechanism of electrochemical reduction of CO_2_ on transition metal–porphyrin complexes: CO_2_ reduction to CO can happen on [FeP]^0^ and [CoP]^0^, whereas HCOO^−^ is the main product on [RhP]^0^ and [IrP]^0^, consistent with experimental observation [27,43,44]. Also consistent with the prediction from this study, hydrogen formation on [FeP]^0^ in a strongly acidic aqueous medium has been reported [27,53]. We note that other factors could affect the stability of reaction intermediates and the chemical potentials of the reactive species, thereby changing the selectivity of the overall reaction. For example, Costentine et al. reported that H_2_ production and CO_2_ reduction in a formic acid buffer on [FeP]^0^ is roughly 50:50 [43]. Costentine et al. also showed that 90% faradaic efficiency for CO can be achieved by maintaining pH = 6.7 via NaOH addition. Based on Equation (2), ΔG°_rxn_(PT) becomes 24.23 kJ/mol at pH 6.7, preventing [FeP-H]^0^ formation while allowing CO_2_ to bind and react with [FeP]^−^ and facilitating CO formation. Although the present study focuses on the thermodynamic aspect of the electrochemical reduction of CO_2_, we note that kinetics plays an important role in determining operating potential and product selectivity [54,55].

## 4. Computational Details

DFT calculations with B3LYP hybrid functional [56] and Grimme’s D3 dispersion corrections [57] have been performed using Gaussian 16 [58]. The Stuttgart–Dresden effective core potentials [59] were adopted for transition metals, and a 6-31g(d,p) basis set was used for the main group elements. The choice of the basis set has been extensively tested against 6-311++G(d,p) in our previous study [20]. Our results showed that the computed reduction potentials using 6-311++G(d,p) and 6-31G(d,p) basis sets differed by less than 50 mV. The same level of theory was used for geometry optimization and frequency analysis. Optimized geometries were confirmed by the absence of any imaginary frequency. The SMD implicit solvation model for water was used [60]. The stability of wavefunctions for all species was also checked. Wavefunction optimization has been performed to include possible unrestricted open-shell diradicals. In this case, single-point energy of a specific spin state, including singlet (doublet), triplet (quartet), quintet (sextet), and septet (octet) was calculated first. These states were then compared, and the lowest-energy spin state was selected for wavefunction optimization. The optimized wavefunctions were used for subsequent geometry optimization and frequency calculations. This strategy allowed us to include possible open-shell radicals’ structures. The spin multiplicities of the complexes and intermediates are presented in Table S1 in the Supplementary Materials.

The methods of determining the reduction potential of the electron transfer steps and pK_a_ value of the carboxylate adducts ([MP-COOH]^0^ and ([MP-COOH]^−^) have been presented in our previous study [20]. The reduction potentials were reported by referencing the standard hydrogen electrode (SHE). The pK_a_ value of [MP-H]^0^ was calculated using the reaction free energy for [MP-H]^0^ → [MP]^−^ + H^+^. The chemical potential of H^+^, G°_solv_(H^+^) = −1125.96 kJ/mol was determined using the solvated proton with six water molecules. The Multiwfn program [61] was used to perform Bader’s atom-in-molecule (AIM) charge [62] analysis.

## 5. Conclusions

A mechanistic analysis of CO_2_ reduction and H_2_ formation on transition metal–porphyrin complex catalysts has been performed based on the results of density functional theory calculations. Reduction of CO_2_ to CO and HCOO^−^ proceeds via the formation of carboxylate adduct ([MP-COOH]^0^ and ([MP-COOH]^−^) and metal-hydride [MP-H]^−^, respectively. The pK_a_ of [MP-H]^0^ determined the pH or buffer acid condition for selectively producing CO or HCOO^−^/H_2_. Furthermore, an ergoneutral pH determined from ΔG°_rxn_(HCOO^−^) defines the lowest pH above which HER will be suppressed. Based on the pK_a_ of [MP-H]^0^, [FeP]^0^ and [CoP]^0^ are selective to CO whereas [RhP]^0^ and [IrP]^0^ can be selective to HCOO^−^. The calculated ergoneutral pH indicates that competing HER can be suppressed on [RhP]^0^ and [IrP]^0^. These findings are in agreement with experimentally observed product selectivities. Furthermore, our study helps to rationalize the experimental observations and provides guidelines to design selective catalysts and choose optimal experimental parameters for CO_2_ reduction.

## Figures and Tables

**Table 1:** The calculated first reduction potentials (E°, V) (Step 1) and second reduction potentials (V) (Step 4) with reference to a standard hydrogen electrode (SHE) for [MP]^0^ catalysts.

Catalyst	First Reduction Potential (E°)	Second Reduction Potential
[FeP]^0^	−1.24	−1.74
[CoP]^0^	−1.28	−1.55
[RhP]^0^	−0.02	−1.64
[IrP]^0^	−0.26	−1.71

**Table 2:** Calculated pK_a_ values of [FeP-H]^0^, [FeP-H]^−^, [CoP-H]^0^, [CoP-H]^−^, [RhP-H]^0^, [RhP-H]^−^, [IrP-H]^0^ and [IrP-H]^−^.

Catalyst	[MP-H]^0^	[MP-H]^−^
[FeP]^0^	2.45	19.45
[CoP]^0^	6.29	13.45
[RhP]^0^	10.07	17.53
[IrP]^0^	11.15	23.75

**Table 3:** Bader charges |e|on the metal center (M) and porphyrin (P) in [MP]^0^ and [MP]^−^. The values in parentheses show the gain of negative charge after one-electron reduction.

Catalyst		[MP]^0^	[MP]^−^
[FeP]^0^	Fe	1.25	1.19 (−0.06)
	P	−1.25	−2.19 (−0.94)
[CoP]^0^	Co	1.17	1.04 (−0.13)
	P	−1.17	−2.04 (−0.87)
[RhP]^0^	Rh	0.89	0.49 (−0.64)
	P	−0.89	−1.49 (−0.36)
[IrP]^0^	Ir	1.13	0.59 (−0.54)
	P	−1.13	−1.60 (−0.46)

## Data Availability

Not applicable.

## Supplementary Materials

The following supporting information can be downloaded at https://www.mdpi.com/article/10.3390/molecules28010375/s1, Figure S1. Electrostatic potential of (a) [FeP]^−^, (b) [CoP]^−^, (c) [RhP]^−^ and (d) [IrP]^−^. (Isovalue for the surfaces: molecular orbitals 0.02 and density 0.004). Table S1. Spin multiplicities of the intermediates.
