# Supplementary material for: Mechanism and Selectivity of Electrochemical Reduction of CO2 on Metalloporphyrin Catalysts from DFT Studies
Source: Molecules. 2023 Jan 2;28(1):375. doi: 10.3390/molecules28010375 (PMC9823635; doi:10.3390/molecules28010375)
Supplement: Supplementary file 1 [file molecules-28-00375-s001.zip › molecules-2102697-supplementary.pdf]

Supporting Information

Mechanism and selectivity of electrochemical reduction of CO_2 on metalloporphyrin catalysts from DFT studies

Zaheer Masood[1] and Qingfeng Ge[1*]

[1] School of Chemical and Biomolecular Sciences, Southern Illinois University Carbondale IL 62901, USA.

*Correspondence: qge@chem.siu.edu

I. Additional data

Table S1. Spin multiplicities of the intermediates

Catalyst	$[MP]^0$	$[MP]^-$	$[MP]^{2-}$	$[MP\text{-}H]^0$	$[MP\text{-}H]^-$	$[MP\text{-}COO]^{2-}$	$[MP\text{-}COO]^-$	$[MP\text{-}COOH]^0$	$[MP\text{-}COOH]^-$	$[MP\text{-}CO]^0$
M = Fe	3**	2	3**	2**	1	2	2	2	1	1
M = Co	2**	1**	2	1	2	2	1	1	2	2
M = Rh	2	1	2	1	2	--	1	--	--	--
M = Ir	2	1	2	1	2	--	1	--	--	--

**open shell diradicals

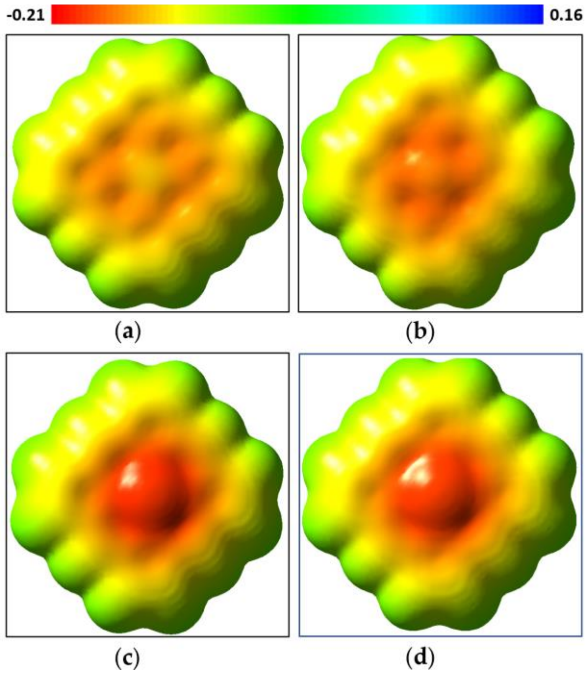

Figure S1. Electrostatic potential of (a) $[FeP]^-$, (b) $[CoP]^-$, (c) $[RhP]^-$ and (d) $[IrP]^-$. (Isovalue for the surfaces: molecular orbitals 0.02 and density 0.004.

II. Relationship between Ergoneutral pH and $\Delta G^{\circ}_{rxn}(HCOO^-)$

$[MP\text{-}H]^- \rightarrow [MP]^0 + H^-$	: $\Delta G^{\circ}(H^-)$, (Hydricity)	(S1)
$H^- + H^+ \rightarrow H_2$	: $\Delta G(H_2) = 2.303RT(pH - pK_{a(H2)})$	(S2)
$[MP\text{-}H]^- + H^+ \rightarrow [MP]^0 + H_2$	: $\Delta G_{rxn}(H_2)$	(S3)
$\Delta G_{rxn}(H_2) = \Delta G^{\circ}(H^-) + 2.303RT \times pH - 2.303RT \times 25.1$		(S4)

In eqn. S4, 25.1 is the pK_a of H_2 [1]. We can related $\Delta G^{\circ}(H^-)$ in eqn. S4 to $\Delta G^{\circ}_{rxn}(HCOO^-)$ based on the following relationships:

$[MP\text{-}H]^- \rightarrow [MP]^0 + H^-$	: $\Delta G^{\circ}(H^-)$, (Hydricity)	(S1)
$H^- + CO_2 \rightarrow HCOO^-$	: -100.74 kJ/mol	(S5)
$[MP\text{-}H]^- + CO_2 \rightarrow [MP]^0 + HCOO^-$	: $\Delta G^{\circ}_{rxn}(HCOO^-)$	(S6)
$\Delta G^{\circ}(H^-) = \Delta G^{\circ}_{rxn}(HCOO^-) + 100.74$ kJ/mol		(S7)

ΔG° for $H^- + CO_2 \rightarrow HCOO^-$ was determined to be -100.74 kJ/mol [1]. By setting $\Delta G_{rxn}(H_2)$ to zero and eliminating $\Delta G^{\circ}(H^-)$ in eqn. S4 using the value of $\Delta G^{\circ}(H^-)$ from eqn. S7, we derived eqn. 6 in the main manuscript that relates the ergoneutral pH to $\Delta G^{\circ}_{rxn}(HCOO^-)$.

III. Optimized structures of the reaction intermediates in Cartesian coordinates

$[CoP]^0$	
**$[CoP]^0$**	**$[CoP]^-$**
Charge Spin Multiplicity	Charge Spin Multiplicity
0 2	0 2
C 0.00000000 0.68097900 -4.21543500	C 0.00000000 0.68097900 -4.2154350
C 0.00000000 0.68097900 4.21543500	C 0.00000000 0.68097900 4.21543500
C 0.00000000 1.09735900 -2.8351070	C 0.00000000 1.09735900 -2.8351070
C 0.00000000 1.09735900 2.83510700	C 0.00000000 1.09735900 2.83510700
C 0.00000000 2.42159800 -2.4215980	C 0.00000000 2.42159800 -2.4215980
C 0.00000000 2.42159800 2.42159800	C 0.00000000 2.42159800 2.42159800
C 0.00000000 2.83510600 -1.0973580	C 0.00000000 2.83510600 -1.0973580
C 0.00000000 2.83510600 1.09735800	C 0.00000000 2.83510600 1.09735800
C 0.00000000 4.21543500 -0.6809790	C 0.00000000 4.21543500 -0.6809790
C 0.00000000 4.21543500 0.68097900	C 0.00000000 4.21543500 0.68097900
C 0.00000000 -0.68097900 -4.2154350	C 0.00000000 -0.68097900 -4.215435
C 0.00000000 -0.68097900 4.21543500	C 0.00000000 -0.68097900 4.2154350
C 0.00000000 -1.09735900 -2.8351070	C 0.00000000 -1.09735900 -2.835107
C 0.00000000 -1.09735900 2.8351070	C 0.00000000 -1.09735900 2.8351070
C 0.00000000 -2.42159800 -2.4215980	C 0.00000000 -2.42159800 -2.421598
C 0.00000000 -2.42159800 2.4215980	C 0.00000000 -2.42159800 2.4215980
C 0.00000000 -2.83510600 -1.0973580	C 0.00000000 -2.83510600 -1.097358
C 0.00000000 -2.83510600 1.0973580	C 0.00000000 -2.83510600 1.0973580
C 0.00000000 -4.21543500 -0.6809790	C 0.00000000 -4.21543500 -0.680979
C 0.00000000 -4.21543500 0.6809790	C 0.00000000 -4.21543500 0.6809790
N 0.00000000 1.99858300 0.00000000	N 0.00000000 1.99858300 0.00000000
N 0.00000000 0.00000000 -1.99858300	N 0.00000000 0.00000000 -1.9985830
N 0.00000000 0.00000000 1.99858300	N 0.00000000 0.00000000 1.99858300
N 0.00000000 -1.99858300 0.00000000	N 0.00000000 -1.99858300 0.0000000
H 0.00000000 1.35929700 -5.05879000	H 0.00000000 1.35929700 -5.0587900

H 0.00000000 1.35929700 5.05879000	H 0.00000000 1.35929700 5.05879000
H 0.00000000 3.18889400 -3.18889100	H 0.00000000 3.18889400 -3.1888910
H 0.00000000 3.18889400 3.18889100	H 0.00000000 3.18889400 3.18889100
H 0.00000000 5.05878600 -1.35930100	H 0.00000000 5.05878600 -1.3593010
H 0.00000000 5.05878600 1.35930100	H 0.00000000 5.05878600 1.35930100
H 0.00000000 -1.35929700 -5.0587900	H 0.00000000 -1.35929700 -5.058790
H 0.00000000 -1.35929700 5.0587900	H 0.00000000 -1.35929700 5.0587900
H 0.00000000 -3.18889400 -3.1888910	H 0.00000000 -3.18889400 -3.188891
H 0.00000000 -3.18889400 3.1888910	H 0.00000000 -3.18889400 3.1888910
H 0.00000000 -5.05878600 -1.3593010	H 0.00000000 -5.05878600 -1.359301
H 0.00000000 -5.05878600 1.3593010	H 0.00000000 -5.05878600 1.3593010
Co 0.00000000 0.00000000 0.00000000	Co 0.00000000 0.00000000 0.0000000
**[CoP-COO]$^{-}$**	**[CoP-COO]$^{2-}$**
Charge Spin Multiplicity	Charge Spin Multiplicity
-1 1	-2 2
N -1.30362900 -1.50563000 -0.289396	N -1.30526667 -1.50764670 -0.31207483
N -1.49384700 1.31852600 -0.3117910	N -1.50811159 1.33138625 -0.30609496
N 1.30356600 1.50563500 -0.28948400	N 1.30508301 1.50733149 -0.31233398
N 1.49377900 -1.31852400 -0.3119830	N 1.50799880 -1.33104546 -0.30651692
C -1.03644400 -2.85869600 -0.275214	C -1.03116662 -2.86853350 -0.29269607
C -2.26070500 -3.62118400 -0.267833	C -2.26885368 -3.62355481 -0.28884915
C -3.28184800 -2.72057500 -0.272065	C -3.28857384 -2.72521749 -0.29722979
C -2.67913800 -1.40997700 -0.281688	C -2.68941776 -1.40544875 -0.29570319
C -3.40293900 -0.22751200 -0.289533	C -3.41959606 -0.23669028 -0.28334575
C -2.84417200 1.04109200 -0.3097250	C -2.86426487 1.05412422 -0.29347409
C -3.61608800 2.25972100 -0.3199990	C -3.62664767 2.25122258 -0.30440702
C -2.72390200 3.28824200 -0.3146450	C -2.72114986 3.29459899 -0.29655955
C -1.40911500 2.69501800 -0.3019250	C -1.42576651 2.71475386 -0.29621787
C -0.22965800 3.42329500 -0.2777520	C -0.22323630 3.44279307 -0.28577710
C 1.03638300 2.85869800 -0.27520000	C 1.03103807 2.86834870 -0.29262544
C 2.26064500 3.62118700 -0.26787500	C 2.26873649 3.62332981 -0.28908763
C 3.28178700 2.72057800 -0.27223400	C 3.28841284 2.72499678 -0.29692763
C 2.67907500 1.40998000 -0.28188500	C 2.68928339 1.40521766 -0.29602566
C 3.40287300 0.22751500 -0.28983500	C 3.41946487 0.23670738 -0.28370121
C 2.84410400 -1.04108900 -0.3100240	C 2.86416126 -1.05392682 -0.29395595
C 3.61602100 -2.25971600 -0.3204030	C 3.62651452 -2.25105303 -0.30491917
C 2.72383800 -3.28823900 -0.3150050	C 2.72101698 -3.29437489 -0.29678614
C 1.40905200 -2.69501900 -0.3021540	C 1.42565048 -2.71453029 -0.29663556
C 0.22959600 -3.42329400 -0.2779020	C 0.22318217 -3.44275107 -0.28599284
H -2.30739400 -4.70252500 -0.265302	H -2.32062736 -4.70545948 -0.29153180
H -4.34883000 -2.90252500 -0.274884	H -4.35657948 -2.90902998 -0.30098429
H -4.48571900 -0.30055800 -0.283599	H -4.50252284 -0.31162971 -0.26705876
H -4.69773500 2.29814000 -0.3321670	H -4.70917438 2.29607613 -0.31827185
H -2.91376300 4.35379900 -0.3205650	H -2.92162851 4.35890105 -0.30115445
H -0.30220900 4.50589800 -0.2659430	H -0.29272062 4.52611199 -0.27742230
H 2.30733400 4.70252800 -0.26528700	H 2.32051169 4.70523410 -0.29183821
H 4.34876900 2.90252800 -0.27512000	H 4.35642605 2.90875907 -0.30038201
H 4.48565400 0.30055900 -0.28398400	H 4.50238672 0.31170010 -0.26732556
H 4.69766700 -2.29813200 -0.33266600	H 4.70903683 -2.29590628 -0.31885004
H 2.91370100 -4.35379500 -0.32097400	H 2.92146855 -4.35868462 -0.30116204
H 0.30214500 -4.50589800 -0.26614800	H 0.29287931 -4.52605598 -0.27752415
Co -0.00002900 -0.00000900 -0.13831700	Co -0.0004152 -0.00004385 -0.10743630
C 0.00002800 -0.00001500 1.82015600	C 0.00034396 0.00005635 1.82891930
O 1.13069200 0.02272100 2.34846100	O 1.12756527 0.00127168 2.38296636
O -1.13057900 -0.02275700 2.34857400	O -1.12647442 -0.00134929 2.38367828

[CoP-COOH]^0
Charge Spin Multiplicity
0 1
N -1.41503500 -1.40079500 -0.32538900
N -1.40016100 1.41588200 -0.32308400
N 1.39984700 1.39856600 -0.31274600
N 1.38499000 -1.41312800 -0.31136700
C -1.24463300 -2.76883500 -0.24663200
C -2.51811800 -3.44074000 -0.23874100
C -3.47268400 -2.47243300 -0.30054300
C -2.78164700 -1.20942300 -0.33682200
C -3.42075200 0.01817400 -0.35219000
C -2.76870800 1.23891500 -0.33374300
C -3.44633900 2.50909100 -0.29503400
C -2.48160400 3.46727500 -0.23331900
C -1.21527400 2.78201600 -0.24361100
C 0.01058000 3.42221000 -0.19489800
C 1.22947000 2.76860800 -0.24196900
C 2.50155200 3.44065600 -0.25724700
C 3.45759500 2.47441400 -0.33479600
C 2.77001100 1.21084700 -0.35724900
C 3.40936500 -0.01793400 -0.38462100
C 2.75703900 -1.23983600 -0.35450600
C 3.43128700 -2.51052100 -0.32901200
C 2.46510200 -3.46662600 -0.25152700
C 1.20011800 -2.78122800 -0.23937800
C -0.02568900 -3.42191900 -0.19503000
H -2.63949300 -4.51507300 -0.19605000
H -4.54940600 -2.57801700 -0.31787900
H -4.50545400 0.02392000 -0.35639200
H -4.52191000 2.62602400 -0.31122200
H -2.59164500 4.54277800 -0.18954200
H 0.01697600 4.50512300 -0.13473300
H 2.62226100 4.51532400 -0.22202700
H 4.53345300 2.58247100 -0.37314500
H 4.49363700 -0.02372000 -0.40625200
H 4.50601100 -2.62989600 -0.36570400
H 2.57450600 -4.54244400 -0.21468100
H -0.03092000 -4.50482200 -0.13456700
Co -0.01350900 0.00010700 -0.19028100
C -0.07451800 -0.00038900 1.69279600
O 1.10725000 -0.00265500 2.36477300
O -1.10238100 0.00109800 2.33885100
H 1.84957500 -0.00351600 1.73973600

[CoP-COOH]$^-$
Charge Spin Multiplicity
-1 2
N -1.42235600 -1.40469000 -0.36976700
N -1.41383800 1.42329600 -0.30449200
N 1.40250600 1.39702100 -0.33862900
N 1.39258000 -1.42973900 -0.30739800
C -1.24964300 -2.78358500 -0.29882100
C -2.53447500 -3.44936300 -0.28796700
C -3.48522000 -2.48261700 -0.31980500
C -2.79767100 -1.20874300 -0.34417900
C -3.43928300 0.00353000 -0.31756400
C -2.78882600 1.24798600 -0.29346900
C -3.45476100 2.50138000 -0.24892200
C -2.47224000 3.47404900 -0.21468100
C -1.22522600 2.79741500 -0.24235000
C 0.02616600 3.43384500 -0.22882900
C 1.22872700 2.77798400 -0.28209000
C 2.51204700 3.44401800 -0.29750000
C 3.46541700 2.47991900 -0.34210300
C 2.78179500 1.20471800 -0.35026900
C 3.42272800 -0.01038300 -0.33643100
C 2.77095300 -1.25684000 -0.31405200
C 3.43443300 -2.50989500 -0.27703000
C 2.45039600 -3.48171400 -0.23420000
C 1.20415500 -2.80493200 -0.24974700
C -0.04782000 -3.44073000 -0.23888900
H -2.65989200 -4.52441100 -0.25752800
H -4.56264700 -2.59001100 -0.32035500
H -4.52441800 0.00482500 -0.30003000
H -4.53024100 2.62571000 -0.24773600
H -2.58640500 4.55015100 -0.18119700
H 0.03820600 4.51814800 -0.18473800
H 2.63635900 4.51946600 -0.27944100
H 4.54215600 2.59026300 -0.36575900
H 4.50770000 -0.01260400 -0.32914500
H 4.50965700 -2.63567500 -0.28516000
H 2.56419200 -4.55797000 -0.20631300
H -0.06021900 -4.52468200 -0.18751900
Co -0.01760500 -0.00578800 -0.18415800
C -0.05383700 -0.02042800 1.68994500
O 1.13997400 0.08777800 2.34485600
O -1.05957400 -0.12139100 2.36891600
H 1.85679500 0.17423700 1.69477200

[CoP-CO]^0
Charge Spin Multiplicity
0 2
N -1.43225700 -1.40972500 -0.31171900
N -1.41725300 1.42472200 -0.31130000
N 1.41753700 1.40839900 -0.29962900
N 1.40255200 -1.42336500 -0.29957800
C -1.25173300 -2.77607800 -0.29319900
C -2.52602700 -3.45097100 -0.29603900
C -3.48453400 -2.48276900 -0.30664100
C -2.79681500 -1.21537300 -0.30923600
C -3.43321900 0.01810600 -0.30405200

[CoP-H]^0
Charge Spin Multiplicity
0 1
C -4.20600500 -0.68073000 -0.00844900
C 4.20600800 -0.68071200 -0.00859500
C -2.82734700 -1.09621900 -0.01682900
C 2.82735200 -1.09620700 -0.01693700
C -2.41917600 -2.41919900 -0.01023000
C 2.41918500 -2.41918900 -0.01031000
C -1.09619600 -2.82732700 -0.01614800
C 1.09620700 -2.82732300 -0.01617800
C -0.68070300 -4.20601000 -0.00690000

C -2.78378900 1.24479500 -0.30877200<br>C -3.45805900 2.51940600 -0.30578500<br>C -2.48938100 3.47742800 -0.29509500<br>C -1.22230900 2.78908300 -0.29259900<br>C 0.01142900 3.42442100 -0.27759500<br>C 1.23797600 2.77418700 -0.29268200<br>C 2.51207400 3.45045600 -0.30991000<br>C 3.47030700 2.48258700 -0.32581600<br>C 2.78155900 1.21520000 -0.31526800<br>C 3.41858700 -0.01808900 -0.31704500<br>C 2.76854600 -1.24458200 -0.31503300<br>C 3.44384100 -2.51919600 -0.32526900<br>C 2.47543900 -3.47689100 -0.30940800<br>C 1.20857300 -2.78717800 -0.29254900<br>C -0.02476800 -3.42441400 -0.27786200<br>H -2.64790000 -4.52633400 -0.29380800<br>H -4.56103600 -2.59381400 -0.31461900<br>H -4.51821600 0.02383500 -0.29891200<br>H -4.53332900 2.64184000 -0.31361800<br>H -2.59985800 4.55401700 -0.29264400<br>H 0.01770600 4.50940000 -0.26566300<br>H 2.63324900 4.52589700 -0.31439300<br>H 4.54672500 2.59315600 -0.34516800<br>H 4.50359800 -0.02381800 -0.32727200<br>H 4.51903500 -2.64115600 -0.34440400<br>H 2.58522300 -4.55355200 -0.31374400<br>H -0.02992800 -4.5093990 -0.26596300<br>Co -0.00621200 -0.000009 -0.18272500<br>C -0.30241900 0.00063900 1.88432800<br>O -0.90920200 0.00196500 2.85065100	C 0.68072000 -4.20600700 -0.00691600<br>C -4.20600700 0.68071200 -0.00844600<br>C 4.20600500 0.68073000 -0.00859300<br>C -2.82735100 1.09620700 -0.01682300<br>C 2.82734700 1.09621900 -0.01693100<br>C -2.41918600 2.41919000 -0.01021500<br>C 2.41917500 2.41920000 -0.01029400<br>C -1.09620800 2.82732300 -0.01612900<br>C 1.09619500 2.82732800 -0.01615900<br>C -0.68072100 4.20600900 -0.00687400<br>C 0.68070200 4.20601200 -0.00688900<br>N 0.00000300 -1.98651600 -0.03190300<br>N -1.98650700 -0.0000040 -0.03211400<br>N 1.98650800 0.00000400 -0.03220300<br>N -0.00000500 1.98651800 -0.03188700<br>H -5.04748100 -1.36112000 -0.00417200<br>H 5.04748700 -1.36109900 -0.00433800<br>H -3.18627300 -3.18629600 0.00261700<br>H 3.18628600 -3.18628200 0.00252100<br>H -1.36113800 -5.04744800 -0.00213700<br>H 1.36115900 -5.04744200 -0.00216900<br>H -5.04748600 1.36110000 -0.00416500<br>H 5.04748100 1.36112100 -0.00433300<br>H -3.18628700 3.18628200 0.00263600<br>H 3.18627300 3.18629600 0.00254100<br>H -1.36116000 5.04744300 -0.00210600<br>H 1.36113800 5.04744900 -0.00213800<br>Co 0.00000100 -0.00000100 0.02647700<br>H 0.00008500 -0.00000900 1.44043200
**[CoP-H]$^{-}$**<br>Charge Spin Multiplicity<br>-1 2<br>C -4.21835100 -0.67836200 -0.02324700<br>C 4.21836200 -0.67831000 -0.03580800<br>C -2.83529200 -1.10522300 -0.02093100<br>C 2.83527900 -1.10514500 -0.03870900<br>C -2.43976800 -2.41919000 0.00240600<br>C 2.43983200 -2.41931200 -0.02824200<br>C -1.10139100 -2.84802900 -0.00185400<br>C 1.10144300 -2.84806700 -0.01963500<br>C -0.69173800 -4.20710800 0.00711800<br>C 0.69189400 -4.20714900 -0.00529900<br>C -4.21833100 0.67833800 -0.03568500<br>C 4.21838200 0.67839000 -0.02335900<br>C -2.83524700 1.10517200 -0.03850800<br>C 2.83532300 1.10525200 -0.02111600<br>C -2.43980000 2.41934100 -0.02809000<br>C 2.43980000 2.41921700 0.00228700<br>C -1.10141100 2.84809700 -0.01954400<br>C 1.10142400 2.84805800 -0.00190400<br>C -0.69186100 4.20717800 -0.00523100<br>C 0.69177200 4.20713800 0.00710000<br>N -0.00007300 -2.00268900 -0.02632500<br>N -1.99048100 -0.00016600 -0.04720700<br>N 1.99051000 0.00019400 -0.04748900	**[CoP]$^{2-}$**<br>Charge Spin Multiplicity<br>-2 2<br>C 0.00000000 0.70088300 -4.23356800<br>C 0.00000000 0.70088300 4.23356800<br>C 0.00000000 1.10475700 -2.88465300<br>C 0.00000000 1.10475700 2.88465300<br>C 0.00000000 2.45370200 -2.42692300<br>C 0.00000000 2.45370200 2.42692300<br>C 0.00000000 2.83347700 -1.11693500<br>C 0.00000000 2.83347700 1.11693500<br>C 0.00000000 4.21914200 -0.67678200<br>C 0.00000000 4.21914200 0.67678200<br>C 0.00000000 -0.70088300 -4.23356800<br>C 0.00000000 -0.70088300 4.23356800<br>C 0.00000000 -1.10475700 -2.88465300<br>C 0.00000000 -1.10475700 2.88465300<br>C 0.00000000 -2.45370200 -2.42692300<br>C 0.00000000 -2.45370200 2.42692300<br>C 0.00000000 -2.83347700 -1.11693500<br>C 0.00000000 -2.83347700 1.11693500<br>C 0.00000000 -4.21914200 -0.67678200<br>C 0.00000000 -4.21914200 0.67678200<br>N 0.00000000 1.97810900 0.00000000<br>N 0.00000000 0.00000000 -2.03997000<br>N 0.00000000 0.00000000 2.03997000

N 0.00010500 2.00271900 -0.02630600	N 0.00000000 -1.97810900 0.00000000
H -5.06257600 -1.35653400 -0.01751200	H 0.00000000 1.37292600 -5.08413500
H 5.06255800 -1.35651700 -0.04159500	H 0.00000000 1.37292600 5.08413500
H -3.21101900 -3.18279900 0.02117000	H 0.00000000 3.23491200 -3.18256300
H 3.21115500 -3.18306400 -0.02260300	H 0.00000000 3.23491200 3.18256300
H -1.36775300 -5.05290700 0.01607700	H 0.00000000 5.06823300 -1.35050500
H 1.36793900 -5.05297100 -0.00689800	H 0.00000000 5.06823300 1.35050500
H -5.06252600 1.35654500 -0.04154000	H 0.00000000 -1.37292600 -5.08413500
H 5.06260600 1.35656200 -0.01754700	H 0.00000000 -1.37292600 5.08413500
H -3.21112300 3.18309300 -0.02247900	H 0.00000000 -3.23491200 -3.18256300
H 3.21105300 3.18282400 0.02108900	H 0.00000000 -3.23491200 3.18256300
H -1.36790600 5.05300000 -0.00678700	H 0.00000000 -5.06823300 -1.35050500
H 1.36778800 5.05293700 0.01602100	H 0.00000000 -5.06823300 1.35050500
Co 0.00002400 0.00001100 0.05294400	Co 0.00000000 0.00000000 0.00000000
H 0.00013100 0.00000000 1.47477800	
[FeP]^0	
**[FeP]^0**	**[FeP]$^-$**
Charge Spin Multiplicity	Charge Spin Multiplicity
0 3	-1 2
N -1.23183300 1.59045100 0.00000000	N -2.03210800 0.00000100 0.00000000
N -1.58780900 -1.23541000 0.00000000	N -0.00000100 -1.99821300 0.00000000
N 1.23181400 -1.59043400 0.00000000	N 2.03210400 -0.00000100 0.00000000
N 1.58779900 1.23541400 0.00000000	N -0.00000100 1.99821300 0.00000000
C -0.87487300 2.92498500 0.00000000	C -2.87010000 1.10220000 0.00000000
C -2.05156200 3.75759400 0.00000000	C -4.23620400 0.69041200 0.00000000
C -3.12738800 2.91978700 0.00000000	C -4.23620300 -0.69041200 0.00000000
C -2.61106100 1.57319600 0.00000000	C -2.87010000 -1.10220000 0.00000000
C -3.39998600 0.42842700 0.00000000	C -2.42779300 -2.43842900 0.00000000
C -2.91972000 -0.87636800 0.00000000	C -1.11005200 -2.84197100 0.00000000
C -3.75398000 -2.05267800 0.00000000	C -0.67970400 -4.22393500 0.00000000
C -2.91974100 -3.13130000 0.00000000	C 0.67969900 -4.22393500 0.00000000
C -1.57325400 -2.61663700 0.00000000	C 1.11004800 -2.84197100 0.00000000
C -0.42920200 -3.40582500 0.00000000	C 2.42778900 -2.43843000 0.00000000
C 0.87485700 -2.92497900 0.00000000	C 2.87009600 -1.10220000 0.00000000
C 2.05154700 -3.75758100 0.00000000	C 4.23620000 -0.69041200 0.00000000
C 3.12737200 -2.91977300 0.00000000	C 4.23620000 0.69041200 0.00000000
C 2.61104500 -1.57318300 0.00000000	C 2.87009600 1.10220000 0.00000000
C 3.39997700 -0.42841800 0.00000000	C 2.42779000 2.43843000 0.00000000
C 2.91970700 0.87637500 0.00000000	C 1.11004900 2.84197100 0.00000000
C 3.75397000 2.05268600 0.00000000	C 0.67970100 4.22393500 0.00000000
C 2.91973100 3.13130600 0.00000000	C -0.67970200 4.22393500 0.00000000
C 1.57324200 2.61664900 0.00000000	C -1.11005100 2.84197000 0.00000000
C 0.42919100 3.40583000 0.00000000	C -2.42779300 2.43843000 0.00000000
H -2.03638800 4.83978400 0.00000000	H -5.08410800 1.36442800 0.00000000
H -4.18007800 3.17162800 0.00000000	H -5.08410800 -1.36442800 0.00000000
H -4.47673100 0.56440300 0.00000000	H -3.18891100 -3.21342000 0.00000000
H -4.83627800 -2.03521300 0.00000000	H -1.35318900 -5.07230600 0.00000000
H -3.17348200 -4.18338400 0.00000000	H 1.35318300 -5.07230700 0.00000000
H -0.56513400 -4.48253500 0.00000000	H 3.18890800 -3.21342000 0.00000000
H 2.03636600 -4.83977100 0.00000000	H 5.08410400 -1.36442800 0.00000000
H 4.18006400 -3.17160200 0.00000000	H 5.08410400 1.36442900 0.00000000
H 4.47672100 -0.56439900 0.00000000	H 3.18890800 3.21342000 0.00000000
H 4.83626800 2.03522300 0.00000000	H 1.35318500 5.07230700 0.00000000
H 3.17346900 4.18339100 0.00000000	H -1.35318700 5.07230700 0.00000000
H 0.56512200 4.48254000 0.00000000	H -3.18891100 3.21342100 0.00000000
Fe 0.00001700 -0.00001000 0.00000000	Fe 0.00000000 0.00000000 0.00000000

**[FePCOO]⁻**<br>Charge Spin Multiplicity<br>-1 2<br>C 5.14614300 1.67811700 0.27877500<br>O 6.01949300 0.97639700 0.82630600<br>O 4.24556400 2.39148500 0.78439500<br>N 3.01022500 1.79052700 -1.95470400<br>N 5.02402400 3.81146100 -1.89949700<br>N 7.01806500 1.81774400 -1.93886300<br>N 5.00436000 -0.20282400 -1.88258200<br>C 2.17092400 0.69464700 -1.96912500<br>C 0.79232700 1.11493600 -2.00365900<br>C 0.79765400 2.47834700 -1.99295900<br>C 2.17930400 2.89065100 -1.95570200<br>C 2.59848900 4.21558100 -1.91908100<br>C 3.92134200 4.63934700 -1.90217500<br>C 4.33031200 6.02379500 -1.91612400<br>C 5.69270400 6.03304400 -1.92898200<br>C 6.11734500 4.65403000 -1.91897600<br>C 7.44196800 4.23972200 -1.94353000<br>C 7.85643800 2.91353000 -1.95814700<br>C 9.23511800 2.49282200 -1.97709800<br>C 9.22974400 1.12933000 -1.95625700<br>C 7.84823000 0.71732900 -1.92351300<br>C 7.42903600 -0.60687400 -1.87784100<br>C 6.10616700 -1.03034500 -1.86641400<br>C 5.69722700 -2.41510500 -1.86953900<br>C 4.33499200 -2.42376000 -1.89131800<br>C 3.91135500 -1.04397200 -1.90141300<br>C 2.58632600 -0.63059400 -1.93887000<br>H -0.05386700 0.44056200 -2.03050700<br>H -0.04445600 3.15834400 -2.00967300<br>H 1.82942200 4.98167000 -1.92177300<br>H 3.64749000 6.86373000 -1.92568600<br>H 6.36482500 6.88115200 -1.95060600<br>H 8.20969900 5.00675800 -1.95681900<br>H 10.08137600 3.16706500 -2.00504800<br>H 10.07198500 0.44933300 -1.96381000<br>H 8.19822700 -1.37273400 -1.86517000<br>H 6.37983900 -3.25526300 -1.86721000<br>H 3.66225300 -3.27146900 -1.90978600<br>H 1.81945500 -1.39856800 -1.94943200<br>Fe 5.01246400 1.81145300 -1.70464500	**[FePCOO]²⁻**<br>Charge Spin Multiplicity<br>-2 1<br>N -1.38733700 -1.44853600 -0.28051100<br>N -1.43685500 1.39865400 -0.28631000<br>N 1.38657400 1.44826200 -0.28243900<br>N 1.43609100 -1.39894200 -0.28716400<br>C -1.18505300 -2.81482900 -0.29229500<br>C -2.44784100 -3.51730700 -0.33224400<br>C -3.42456300 -2.56661000 -0.34619100<br>C -2.75800600 -1.28420700 -0.31361900<br>C -3.41633500 -0.05947600 -0.32163000<br>C -2.80085000 1.18731400 -0.32299300<br>C -3.51112200 2.44571300 -0.36344900<br>C -2.56784300 3.42958200 -0.34879200<br>C -1.28173400 2.77118600 -0.30094300<br>C -0.05998000 3.43446900 -0.28710400<br>C 1.18428200 2.81455500 -0.29453700<br>C 2.44702700 3.51701200 -0.33601200<br>C 3.42373300 2.56630700 -0.35064000<br>C 2.75720800 1.28391800 -0.31689300<br>C 3.41552600 0.05918200 -0.32513500<br>C 2.80004300 -1.18761000 -0.32541400<br>C 3.51027100 -2.44601800 -0.36628200<br>C 2.56701000 -3.42988700 -0.35022800<br>C 1.28095400 -2.77148200 -0.30119800<br>C 0.05921100 -3.43475200 -0.28589200<br>H -2.55177400 -4.59506600 -0.35536100<br>H -4.49864600 -2.70065900 -0.38380100<br>H -4.50225500 -0.07838400 -0.34752800<br>H -4.58903500 2.54254500 -0.40527700<br>H -2.70863300 4.50307100 -0.37568000<br>H -0.07904500 4.52048700 -0.29847500<br>H 2.55094100 4.59476200 -0.35962500<br>H 4.49777700 2.70034100 -0.38940600<br>H 4.50141800 0.07808600 -0.35221700<br>H 4.58813400 -2.54286600 -0.40932900<br>H 2.70778300 -4.50338300 -0.37694600<br>H 0.07825300 -4.52077400 -0.29690300<br>Fe -0.00032200 -0.00009800 -0.07952100<br>C 0.00019200 0.00026400 1.85737500<br>O 1.11907000 0.00641300 2.49089200<br>O -1.11826500 -0.00567400 2.49154900
**[FePCOOH⁰**<br>Charge Spin Multiplicity<br>0 2<br>N -1.43182200 -1.41114000 -0.32366600<br>N -1.41690800 1.42639000 -0.32275800<br>N 1.39498700 1.41134800 -0.30676000<br>N 1.38005700 -1.42598700 -0.30601300<br>C -1.25989600 -2.78317300 -0.29674100<br>C -2.53455800 -3.45114100 -0.28354300<br>C -3.48867100 -2.47844300 -0.28660900	**[FePCOOH]⁻**<br>Charge Spin Multiplicity<br>-1 1<br>N -1.39413100 -1.44383900 -0.32217600<br>N -1.44382400 1.39762600 -0.31821800<br>N 1.39509900 1.44704200 -0.31234900<br>N 1.44428800 -1.39292900 -0.30400500<br>C -1.18592000 -2.80669400 -0.30498600<br>C -2.44698600 -3.51334100 -0.32575900<br>C -3.42612100 -2.56629500 -0.34972400

C -2.79791800 -1.21460600 -0.30390900<br>C -3.43292200 0.01816900 -0.29448100<br>C -2.78498000 1.24421200 -0.30281500<br>C -3.46236100 2.51527500 -0.28471200<br>C -2.49809000 3.47790200 -0.28161800<br>C -1.23053500 2.79653100 -0.29559900<br>C -0.00093800 3.43532500 -0.28192700<br>C 1.22235900 2.78323500 -0.29276700<br>C 2.49636800 3.45178900 -0.29268600<br>C 3.45265300 2.48098400 -0.30001600<br>C 2.76403100 1.21686900 -0.30253400<br>C 3.39926200 -0.01792900 -0.29576100<br>C 2.75099300 -1.24593900 -0.30141800<br>C 3.42628700 -2.51725200 -0.29798700<br>C 2.45987500 -3.47796300 -0.29069600<br>C 1.19298600 -2.79595800 -0.29174300<br>C -0.03715300 -3.43500700 -0.28199400<br>H -2.66051000 -4.52573100 -0.27314500<br>H -4.56551400 -2.58476300 -0.27872800<br>H -4.51765400 0.02385700 -0.27711900<br>H -4.53802800 2.63292900 -0.27650400<br>H -2.61274700 4.55374600 -0.27085200<br>H 0.00530800 4.51991200 -0.26276800<br>H 2.62110500 4.52656900 -0.29319000<br>H 4.52925400 2.58889000 -0.30750700<br>H 4.48380300 -0.02364100 -0.28148600<br>H 4.50170300 -2.63647300 -0.30502000<br>H 2.57328800 -4.55399100 -0.29077800<br>H -0.04251900 -4.51959800 -0.26280400<br>C 0.03023900 -0.00042900 1.75066200<br>O 1.21258300 -0.00317700 2.41757700<br>O -1.00022000 0.00169700 2.40138700<br>H 1.95103000 -0.00399200 1.78737000<br>Fe -0.03232700 0.00026100 -0.16913600	C -2.76286300 -1.28170200 -0.34116200<br>C -3.42292800 -0.05790200 -0.34252200<br>C -2.80617500 1.18813200 -0.33556100<br>C -3.51358500 2.44896400 -0.34029600<br>C -2.56791600 3.42939100 -0.31743000<br>C -1.28285100 2.76712000 -0.30119000<br>C -0.05963700 3.42782800 -0.28181400<br>C 1.18608100 2.81101800 -0.30188100<br>C 2.44631100 3.51728800 -0.33243300<br>C 3.42712100 2.57173900 -0.35962500<br>C 2.76649700 1.28694800 -0.34403200<br>C 3.42650300 0.06158800 -0.34479100<br>C 2.80890800 -1.18540100 -0.33319200<br>C 3.51427500 -2.44638800 -0.34027000<br>C 2.56716300 -3.42552800 -0.31230500<br>C 1.28281000 -2.76342200 -0.28989800<br>C 0.05961600 -3.42389800 -0.27695600<br>H -2.54729000 -4.59138300 -0.32650800<br>H -4.49989700 -2.70317000 -0.37422500<br>H -4.50866400 -0.07679900 -0.35344600<br>H -4.59149700 2.54854300 -0.36249900<br>H -2.70559000 4.50330800 -0.31701000<br>H -0.07852100 4.51350100 -0.27088500<br>H 2.54582100 4.59537200 -0.33965000<br>H 4.50040300 2.71007900 -0.39242200<br>H 4.51199000 0.08013300 -0.35717000<br>H 4.59183300 -2.54716700 -0.36954100<br>H 2.70411700 -4.49952700 -0.31492200<br>H 0.07888900 -4.50954500 -0.26436600<br>Fe -0.00552900 0.00210700 -0.14795400<br>C -0.05237600 0.00314500 1.73468800<br>O 1.16619100 0.07104200 2.43208600<br>O -1.03442600 -0.05021600 2.48714500<br>H 1.87000900 0.11686000 1.76727800
**[FePCO]^0**<br>Charge Spin Multiplicity<br>0 1<br>N -1.46159900 -1.41018900 -0.31575300<br>N -1.44658500 1.42537000 -0.31545900<br>N 1.38862300 1.41041600 -0.28885800<br>N 1.37362800 -1.42529600 -0.28897100<br>C -1.28260300 -2.77841600 -0.30101200<br>C -2.55745100 -3.45263900 -0.31513300<br>C -3.51555800 -2.48423200 -0.32589500<br>C -2.82783400 -1.21657000 -0.31800800<br>C -3.46200000 0.01818800 -0.31531600<br>C -2.81479500 1.24618300 -0.31771700<br>C -3.48907200 2.52106200 -0.32534400<br>C -2.52079300 3.47929600 -0.31445900<br>C -1.25315800 2.79161200 -0.30054000<br>C -0.01855400 3.42571600 -0.28070100<br>C 1.20944400 2.77854800 -0.28140900<br>C 2.48445500 3.45281800 -0.28172600<br>C 3.44256500 2.48438900 -0.27806500<br>C 2.75468000 1.21670900 -0.27469500	**[FeP-H]^0**<br>Charge Spin Multiplicity<br>0 2<br>N -1.43272700 1.39457300 -0.03295600<br>N 1.40675200 1.42041500 -0.03274700<br>N 1.42998800 -1.39461200 -0.02656200<br>N -1.40398500 -1.42041000 -0.02619900<br>C -2.80119400 1.20283800 -0.05719900<br>C -3.48854000 2.46763400 -0.04027000<br>C -2.53020100 3.43563800 0.00898500<br>C -1.25619600 2.76311900 0.01704800<br>C -0.03129400 3.41460300 0.04901300<br>C 1.20525400 2.78561500 0.01695100<br>C 2.46679700 3.48126700 0.00880000<br>C 3.44257500 2.53087100 -0.04016500<br>C 2.77845300 1.25371600 -0.05721000<br>C 3.43407500 0.03108400 -0.07228800<br>C 2.80047500 -1.20189100 -0.04984300<br>C 3.48768700 -2.46640400 -0.03585600<br>C 2.52994600 -3.43512300 0.00954500<br>C 1.25536700 -2.76425600 0.01523600

C 3.38875400 -0.01803100 -0.26394100<br>C 2.74166100 -1.24601400 -0.27475400<br>C 3.41608900 -2.52091800 -0.27816200<br>C 2.44780500 -3.47917100 -0.28189900<br>C 1.18001000 -2.79144500 -0.28161100<br>C -0.05476200 -3.42555900 -0.28108100<br>H -2.68038400 -4.52786200 -0.32079600<br>H -4.59191700 -2.59573000 -0.34205600<br>H -4.54719900 0.02391300 -0.31591400<br>H -4.56419600 2.64395000 -0.34142600<br>H -2.63232400 4.55575900 -0.31996300<br>H -0.01290700 4.51087900 -0.27129300<br>H 2.60752900 4.52802900 -0.28995000<br>H 4.51905600 2.59576800 -0.28261400<br>H 4.47389700 -0.02374400 -0.25274900<br>H 4.49134500 -2.64369300 -0.28268300<br>H 2.55949400 -4.55561900 -0.29017200<br>H -0.06058500 -4.51072300 -0.27174800<br>C -0.05436200 0.00002500 1.59477900<br>O -0.06524700 0.00001200 2.74966400<br>Fe -0.03828000 0.00004400 -0.13362600	C 0.03135400 -3.41713300 0.04213800<br>C -1.20442000 -2.78665000 0.01545500<br>C -2.46643800 -3.48076200 0.00934700<br>C -3.44165200 -2.52970000 -0.03589400<br>C -2.77783900 -1.25282900 -0.04968600<br>C -3.43410100 -0.03166900 -0.07223800<br>H -4.56466300 2.57911000 -0.06228800<br>H -2.65369600 4.51051400 0.03328000<br>H -0.04126400 4.49898100 0.08682100<br>H 2.57072900 4.55821000 0.03301300<br>H 4.51650500 2.66185000 -0.06203700<br>H 4.51890900 0.04056100 -0.08800500<br>H 4.56389600 -2.57735400 -0.05625700<br>H 2.65424700 -4.50992400 0.03304500<br>H 0.04126500 -4.50166900 0.07295800<br>H -2.57116200 -4.55764700 0.03249200<br>H -4.51565400 -2.66028200 -0.05652100<br>H -4.51892500 -0.04237900 -0.08827400<br>H -0.00085400 0.10933000 1.52099300<br>Fe -0.00000700 -0.00418700 0.05149300
**[FeP-H]$^{-}$**<br>Charge Spin Multiplicity<br>-1 1<br>N -2.00853100 -0.00967800 0.00028200<br>N -0.00959000 2.00841300 0.00042900<br>N 2.00833300 0.00965300 -0.00006400<br>N 0.00974900 -2.00840200 0.00040900<br>C -2.83998100 -1.11188500 -0.02355700<br>C -4.22535900 -0.70206000 -0.06294700<br>C -4.23213000 0.66067900 -0.06296200<br>C -2.85084900 1.08416000 -0.02351100<br>C -2.43497200 2.41137600 -0.02223200<br>C -1.11188700 2.83983100 -0.02323900<br>C -0.70208200 4.22526300 -0.06259900<br>C 0.66062300 4.23201100 -0.06203100<br>C 1.08413300 2.85067300 -0.02333900<br>C 2.41134600 2.43465600 -0.02236700<br>C 2.83986400 1.11161600 -0.02359300<br>C 4.22539000 0.70188700 -0.06244300<br>C 4.23217100 -0.66049100 -0.06241800<br>C 2.85075700 -1.08391300 -0.02356700<br>C 2.43503700 -2.41097300 -0.02237800<br>C 1.11184000 -2.83979500 -0.02338600<br>C 0.70201300 -4.22523500 -0.06205100<br>C -0.66068300 -4.23204200 -0.06261400<br>C -1.08417700 -2.85071700 -0.02322700<br>C -2.41127500 -2.43508000 -0.02225200<br>H -5.06653600 -1.38328100 -0.09406300<br>H -5.08012300 1.33337500 -0.09409900<br>H -3.20655900 3.17549900 -0.04167700<br>H -1.38319700 5.06654100 -0.09373000<br>H 1.33323900 5.08006300 -0.09270900<br>H 3.17548200 3.20625100 -0.04153900<br>H 5.06688200 1.38270200 -0.09317800	**[FeP]$^{2-}$**<br>Charge Spin Multiplicity<br>-2 1<br>N 2.03866200 0.00000000 0.00000000<br>N -0.00001600 -1.95036500 0.00000000<br>N -2.03861100 0.00000000 0.00000000<br>N -0.00001600 1.95036500 0.00000000<br>C 2.88503300 1.10054700 0.00000000<br>C 4.24507200 0.69435700 0.00000000<br>C 4.24507200 -0.69435700 0.00000000<br>C 2.88503300 -1.10054700 0.00000000<br>C 2.42933200 -2.43750900 0.00000000<br>C 1.11519800 -2.81835100 0.00000000<br>C 0.67738600 -4.20179700 0.00000000<br>C -0.67735900 -4.20181500 0.00000000<br>C -1.11521300 -2.81839200 0.00000000<br>C -2.42933500 -2.43752500 0.00000000<br>C -2.88499400 -1.10054300 0.00000000<br>C -4.24502900 -0.69435900 0.00000000<br>C -4.24502900 0.69435900 0.00000000<br>C -2.88499400 1.10054300 0.00000000<br>C -2.42933500 2.43752500 0.00000000<br>C -1.11521300 2.81839200 0.00000000<br>C -0.67735900 4.20181500 0.00000000<br>C 0.67738600 4.20179700 0.00000000<br>C 1.11519800 2.81835100 0.00000000<br>C 2.42933200 2.43750900 0.00000000<br>H 5.09521800 1.36679500 0.00000000<br>H 5.09521800 -1.36679500 0.00000000<br>H 3.18196500 -3.22205600 0.00000000<br>H 1.35153800 -5.05046500 0.00000000<br>H -1.35148400 -5.05050600 0.00000000<br>H -3.18201100 -3.22202900 0.00000000<br>H -5.09515400 -1.36682200 0.00000000

<table>
<tr><td>H 5.08049000 -1.33275900 -0.09315400
H 3.20661600 -3.17512800 -0.04154700
H 1.38317100 -5.06645900 -0.09272200
H -1.33326500 -5.08014300 -0.09375100
H -3.17540000 -3.20666700 -0.04169500
H 0.00005600 0.00001500 1.68614600
Fe 0.00002900 0.00001300 0.14925400</td><td>H -5.09515400 1.36682200 0.00000000
H -3.18201100 3.22202900 0.00000000
H -1.35148400 5.05050600 0.00000000
H 1.35153800 5.05046500 0.00000000
H 3.18196500 3.22205600 0.00000000
Fe -0.00005300 0.00000000 0.00000000</td></tr>
<tr><td colspan="2">$[RhP]^0$</td></tr>
<tr><td>$[RhP]^0$
Charge Spin Multiplicity
0 2
N -2.03724900 0.00000000 0.00000000
N 0.00016200 2.05659300 0.00000000
N 2.03709700 0.00000000 0.00000000
N 0.00016200 -2.05659300 0.00000000
C -2.86053700 -1.11263200 0.00000000
C -4.23066300 -0.68553900 0.00000000
C -4.23066300 0.68553900 0.00000000
C -2.86053700 1.11263200 0.00000000
C -2.42798400 2.43984200 0.00000000
C -1.10645800 2.87576900 0.00000000
C -0.68079500 4.26011600 0.00000000
C 0.68069600 4.26031200 0.00000000
C 1.10630000 2.87608600 0.00000000
C 2.42795100 2.43996900 0.00000000
C 2.86023400 1.11268900 0.00000000
C 4.23052200 0.68529200 0.00000000
C 4.23052200 -0.68529200 0.00000000
C 2.86023400 -1.11268900 0.00000000
C 2.42795100 -2.43996900 0.00000000
C 1.10630000 -2.87608600 0.00000000
C 0.68069600 -4.26031200 0.00000000
C -0.68079500 -4.26011600 0.00000000
C -1.10645800 -2.87576900 0.00000000
C -2.42798400 -2.43984200 0.00000000
H -5.08120300 -1.35477200 0.00000000
H -5.08120300 1.35477200 0.00000000
H -3.19895300 3.20394800 0.00000000
H -1.35524700 5.10651800 0.00000000
H 1.35508200 5.10675000 0.00000000
H 3.19883400 3.20417600 0.00000000
H 5.08144600 1.35401400 0.00000000
H 5.08144600 -1.35401400 0.00000000
H 3.19883400 -3.20417600 0.00000000
H 1.35508200 -5.10675000 0.00000000
H -1.35524700 -5.10651800 0.00000000
H -3.19895300 -3.20394800 0.00000000
Rh 0.00017100 0.00000000 0.00000000</td><td>$[RhP]^-$
Charge Spin Multiplicity
-1 1
N -2.04145900 0.00000000 0.00000000
N 0.00013100 2.04115600 0.00000000
N 2.04123900 0.00000000 0.00000000
N 0.00013100 -2.04115600 0.00000000
C -2.86748100 -1.10750200 0.00000000
C -4.24955100 -0.68204800 0.00000000
C -4.24955100 0.68204800 0.00000000
C -2.86748100 1.10750300 0.00000000
C -2.43158300 2.43143500 0.00000000
C -1.10760800 2.86706300 0.00000000
C -0.68178200 4.24941400 0.00000000
C 0.68210600 4.24957200 0.00000000
C 1.10752100 2.86726800 0.00000000
C 2.43145700 2.43139800 0.00000000
C 2.86706500 1.10766100 0.00000000
C 4.24954700 0.68188700 0.00000000
C 4.24954700 -0.68188700 0.00000000
C 2.86706500 -1.10766100 0.00000000
C 2.43145700 -2.43139800 0.00000000
C 1.10752100 -2.86726800 0.00000000
C 0.68210600 -4.24957200 0.00000000
C -0.68178200 -4.24941400 0.00000000
C -1.10760800 -2.86706300 0.00000000
C -2.43158300 -2.43143500 0.00000000
H -5.09791400 -1.35493000 0.00000000
H -5.09791400 1.35493000 0.00000000
H -3.19999600 3.19941500 0.00000000
H -1.35453000 5.09789300 0.00000000
H 1.35495000 5.09797500 0.00000000
H 3.19962200 3.19965400 0.00000000
H 5.09838100 1.35420500 0.00000000
H 5.09838100 -1.35420500 0.00000000
H 3.19962200 -3.19965500 0.00000000
H 1.35495000 -5.09797600 0.00000000
H -1.35453100 -5.09789300 0.00000000
H -3.19999600 -3.19941500 0.00000000
Rh 0.00005300 0.00000000 0.00000000</td></tr>
<tr><td>$[RhP\text{-}H]^0$
Charge Spin Multiplicity
0 1
N 0.00894700 2.05181500 -0.03009800
N 2.05186000 -0.00894800 -0.03001200
N -0.00894600 -2.05184800 -0.02998200
N -2.05186000 0.00894300 -0.03001600</td><td>$[RhP\text{-}H]^-$
Charge Spin Multiplicity
-1 2
N 0.00000200 2.06694900 -0.02855000
N 2.06264400 -0.00000200 -0.03469700
N -0.00000200 -2.06694500 -0.02853700
N -2.06264400 0.00000200 -0.03469700</td></tr>
</table>

C -1.09570800 2.87323200 -0.00939400<br>C -0.66328200 4.25207500 0.00968500<br>C 0.70065000 4.24605700 0.00964700<br>C 1.12084200 2.86346600 -0.00942400<br>C 2.44271800 2.42135600 -0.00275200<br>C 2.87331900 1.09568900 -0.00941200<br>C 4.25212600 0.66327600 0.00961300<br>C 4.24605600 -0.70066300 0.00967600<br>C 2.86351500 -1.12084100 -0.00931400<br>C 2.42145400 -2.44271600 -0.00258600<br>C 1.09572100 -2.87325800 -0.00936200<br>C 0.66329700 -4.25201000 0.00949300<br>C -0.70065400 -4.24599500 0.00948500<br>C -1.12085200 -2.86349300 -0.00936600<br>C -2.44277600 -2.42139600 -0.00257900<br>C -2.87328200 -1.09570900 -0.00930000<br>C -4.25207600 -0.66329900 0.00967500<br>C -4.24610800 0.70064000 0.00958700<br>C -2.86355300 1.12081900 -0.00942500<br>C -2.42139500 2.44267700 -0.00273400<br>H -1.33284900 5.10230900 0.02137300<br>H 1.37770600 5.09036400 0.02135000<br>H 3.21357500 3.18554000 0.01472700<br>H 5.10232900 1.33287200 0.02127000<br>H 5.09032000 -1.37777900 0.02142100<br>H 3.18568400 -3.21351800 0.01493900<br>H 1.33300700 -5.10211700 0.02118500<br>H -1.37784900 -5.09017700 0.02121400<br>H -3.21358600 -3.18561700 0.01495700<br>H -5.10226800 -1.33292600 0.02138200<br>H -5.09038300 1.37772600 0.02127000<br>H -3.18567200 3.21344200 0.01475800<br>Rh -0.00000200 0.00000200 -0.01829000<br>H -0.00000500 0.00065100 1.48670500	C -1.11331900 2.88797300 -0.01151400<br>C -0.69334500 4.24904400 0.00213200<br>C 0.69335400 4.24904300 0.00213200<br>C 1.11332500 2.88797100 -0.01151400<br>C 2.45393400 2.43203400 -0.00542100<br>C 2.87960000 1.11695600 -0.01351500<br>C 4.26438800 0.68019500 0.00125200<br>C 4.26438700 -0.68020200 0.00125700<br>C 2.87959900 -1.11696000 -0.01351000<br>C 2.45392800 -2.43203700 -0.00540900<br>C 1.11331900 -2.88797000 -0.01149900<br>C 0.69334500 -4.24904000 0.00215400<br>C -0.69335400 -4.24903900 0.00215400<br>C -1.11332500 -2.88796700 -0.01149900<br>C -2.45393300 -2.43203200 -0.00540900<br>C -2.87960100 -1.11695400 -0.01351000<br>C -4.26438900 -0.68019300 0.00125700<br>C -4.26438700 0.68020400 0.00125200<br>C -2.87959800 1.11696100 -0.01351500<br>C -2.45392900 2.43203900 -0.00542100<br>H -1.36194500 5.10098600 0.00934600<br>H 1.36195600 5.10098300 0.00934600<br>H 3.22443700 3.19752400 0.01018700<br>H 5.11496500 1.35061400 0.00991200<br>H 5.11496200 -1.35062300 0.00992300<br>H 3.22443100 -3.19752800 0.01020300<br>H 1.36194400 -5.10098300 0.00937100<br>H -1.36195400 -5.10098000 0.00937100<br>H -3.22443700 -3.19752200 0.01020300<br>H -5.11496500 -1.35061200 0.00992300<br>H -5.11496200 1.35062500 0.00991300<br>H -3.22443100 3.19753000 0.01018700<br>Rh 0.00000000 -0.00000400 -0.00201400<br>H 0.00000000 -0.00000900 1.50698100
**[RhP]$^{2-}$**<br>Charge Spin Multiplicity<br>-2 2<br>N 1.77152100 1.01397100 -0.00025800<br>N 3.82870200 3.07110200 -0.00023300<br>N 5.88594400 1.01394100 -0.00025800<br>N 3.82872800 -1.04317500 -0.00024300<br>C 0.94769000 -0.10118100 -0.00009500<br>C -0.43818100 0.33302800 0.00008300<br>C -0.43817700 1.69485700 0.00005700<br>C 0.94773200 2.12907600 -0.00009400<br>C 1.37533700 3.44607500 -0.00006700<br>C 2.71587700 3.90102100 -0.00008000<br>C 3.13540000 5.26476000 0.00010600<br>C 4.52209200 5.26474600 0.00010600<br>C 4.94161200 3.90103700 -0.00007900<br>C 6.28210200 3.44605700 -0.00006600<br>C 6.70974300 2.12910500 -0.00009400<br>C 8.09563100 1.69490300 0.00005600<br>C 8.09563400 0.33306800 0.00008500<br>C 6.70972800 -0.10114400 -0.00009400	

C 6.28210700 -1.41815600 -0.00006200<br>C 4.94157300 -1.87307900 -0.00007900<br>C 4.52203200 -3.23682300 0.00009400<br>C 3.13534700 -3.23682400 0.00009500<br>C 2.71582400 -1.87311100 -0.00007900<br>C 1.37534200 -1.41813900 -0.00006200<br>H -1.29011600 -0.33685800 0.00021700<br>H -1.29011100 2.36474400 0.00016900<br>H 0.60424500 4.21271600 0.00008300<br>H 2.46667200 6.11765000 0.00024100<br>H 5.19081800 6.11763400 0.00024100<br>H 7.05319400 4.21270300 0.00008300<br>H 8.94757200 2.36478000 0.00016700<br>H 8.94757200 -0.33681600 0.00021900<br>H 7.05321100 -2.18478400 0.00009400<br>H 5.19073900 -4.08972600 0.00022600<br>H 2.46663900 -4.08972900 0.00022700<br>H 0.60423500 -2.18477300 0.00009300<br>Rh 3.82876000 1.01395600 -0.00079900	
**[IrP]^0**	
**[IrP]^0**<br>Charge Spin Multiplicity<br>0 2<br>N -2.03656100 0.00000000 0.00000000<br>N 0.00015500 2.05804200 0.00000000<br>N 2.03643000 0.00000000 0.00000000<br>N 0.00015500 -2.05804200 0.00000000<br>C -2.86192000 -1.11606900 0.00000000<br>C -4.22752100 -0.68650100 0.00000000<br>C -4.22752100 0.68650000 0.00000000<br>C -2.86192000 1.11606900 0.00000000<br>C -2.42761000 2.44335500 0.00000000<br>C -1.10920000 2.88008500 0.00000000<br>C -0.68018700 4.26389400 0.00000000<br>C 0.67985900 4.26414000 0.00000000<br>C 1.10898700 2.88049400 0.00000000<br>C 2.42756400 2.44352900 0.00000000<br>C 2.86159800 1.11615200 0.00000000<br>C 4.22734800 0.68628500 0.00000000<br>C 4.22734800 -0.68628500 0.00000000<br>C 2.86159800 -1.11615200 0.00000000<br>C 2.42756400 -2.44352900 0.00000000<br>C 1.10898700 -2.88049400 0.00000000<br>C 0.67985900 -4.26414000 0.00000000<br>C -0.68018700 -4.26389400 0.00000000<br>C -1.10920000 -2.88008500 0.00000000<br>C -2.42761000 -2.44335500 0.00000000<br>H -5.07913000 -1.35421800 0.00000000<br>H -5.07913000 1.35421800 0.00000000<br>H -3.19927400 3.20675700 0.00000000<br>H -1.35451500 5.11029500 0.00000000<br>H 1.35403300 5.11064500 0.00000000<br>H 3.19918600 3.20699300 0.00000000<br>H 5.07928100 1.35357100 0.00000000<br>H 5.07928100 -1.35357100 0.00000000	**[IrP]$^-$**<br>Charge Spin Multiplicity<br>-1 1<br>N -1.97702731 0.00001091 0.00000000<br>N 0.00101384 2.01331405 0.00000000<br>N 1.99710998 0.00000091 0.00000000<br>N 0.00101584 -2.01331924 0.00000000<br>C -2.87640337 -1.15673717 0.00000000<br>C -4.33422647 -0.67182014 0.00000000<br>C -4.33422647 0.67183795 0.00000000<br>C -2.87640337 1.15675599 0.00000000<br>C -2.52179334 2.42875208 0.00000000<br>C -1.05736024 2.81738311 0.00000000<br>C -0.63950321 4.30239821 0.00000000<br>C 0.68491989 4.36265422 0.00000000<br>C 1.18008492 2.92595112 0.00000000<br>C 2.47113101 2.61245510 0.00000000<br>C 2.89212204 1.16896599 0.00000000<br>C 4.17778214 0.76000296 0.00000000<br>C 4.17778014 -0.75999615 0.00000000<br>C 2.89212204 -1.16896218 0.00000000<br>C 2.47113302 -2.61245428 0.00000000<br>C 1.18008592 -2.92595330 0.00000000<br>C 0.68491789 -4.36265441 0.00000000<br>C -0.63950721 -4.30239640 0.00000000<br>C -1.05736024 -2.81738029 0.00000000<br>C -2.52179334 -2.42873927 0.00000000<br>H -5.20205654 -1.29773419 0.00000000<br>H -5.20205954 1.29775300 0.00000000<br>H -3.27366240 3.19006114 0.00000000<br>H -1.30478326 5.14043228 0.00000000<br>H 1.28618193 5.24774429 0.00000000<br>H 3.21030407 3.38609715 0.00000000<br>H 5.04148220 1.39160801 0.00000000<br>H 5.04147820 -1.39160019 0.00000000

H 3.19918600 -3.20699300 0.00000000<br>H 1.35403300 -5.11064500 0.00000000<br>H -1.35451500 -5.11029500 0.00000000<br>H -3.19927400 -3.20675700 0.00000000<br>Ir 0.00016300 0.00000000 0.00000000	H 3.21030706 -3.38609734 0.00000000<br>H 1.28617793 -5.24774547 0.00000000<br>H -1.30479026 -5.14042846 0.00000000<br>H -3.27366440 -3.19004632 0.00000000<br>Ir 0.00791284 -0.00000609 0.00000000
**[IrP-H]^{0}**<br>Charge Spin Multiplicity<br>0 1<br>N 2.04731400 -0.23170200 -0.02409400<br>N -0.23168400 -2.04730900 -0.02400300<br>N -2.04736500 0.23168500 -0.02432600<br>N 0.23170100 2.04734200 -0.02433800<br>C 2.98231700 0.78116100 -0.00342500<br>C 4.30526600 0.19924700 0.01947100<br>C 4.15200000 -1.15605500 0.01947200<br>C 2.73246400 -1.42770700 -0.00333000<br>C 2.14527800 -2.69257500 0.00346000<br>C 0.78116400 -2.98231900 -0.00323500<br>C 0.19924100 -4.30526300 0.01973000<br>C -1.15606000 -4.15198900 0.01967800<br>C -1.42770300 -2.73245300 -0.00324600<br>C -2.69257800 -2.14528400 0.00344100<br>C -2.98236400 -0.78117500 -0.00340600<br>C -4.30530400 -0.19929400 0.01967000<br>C -4.15201600 1.15610900 0.01958400<br>C -2.73249200 1.42771100 -0.00346900<br>C -2.14527300 2.69257500 0.00329200<br>C -0.78116400 2.98233300 -0.00348100<br>C -0.19924900 4.30528000 0.01959500<br>C 1.15605800 4.15201100 0.01953800<br>C 1.42770300 2.73247600 -0.00347500<br>C 2.69257000 2.14527600 0.00328400<br>H 5.22373100 0.77161000 0.03474800<br>H 4.91929400 -1.91914500 0.03448000<br>H 2.82196000 -3.54142200 0.02216500<br>H 0.77160100 -5.22372800 0.03509800<br>H -1.91915600 -4.91927600 0.03472300<br>H -3.54141200 -2.82198100 0.02223300<br>H -5.22377200 -0.77164400 0.03514200<br>H -4.91930800 1.91919700 0.03471300<br>H -2.82195100 3.54142300 0.02211500<br>H -0.77161100 5.22374200 0.03505000<br>H 1.91915100 4.91930000 0.03466700<br>H 3.54142000 2.82195300 0.02201400<br>H -0.00019000 0.00028700 1.51286600<br>Ir 0.00001700 -0.00001000 -0.02677400	**[IrP-H]$^{-}$**<br>Charge Spin Multiplicity<br>-1 2<br>N -0.00000500 -2.07583200 -0.02400200<br>N -2.07488700 0.00001400 -0.02850400<br>N 0.00000500 2.07582700 -0.02435100<br>N 2.07488700 0.00000400 -0.02850400<br>C 1.11645200 -2.89503400 -0.00788400<br>C 0.69333700 -4.25586000 0.00843100<br>C -0.69335700 -4.25585700 0.00843100<br>C -1.11646500 -2.89502800 -0.00788400<br>C -2.45635900 -2.43435100 -0.00136100<br>C -2.88854100 -1.11970200 -0.00880600<br>C -4.27267900 -0.68048600 0.00872200<br>C -4.27268100 0.68049900 0.00874600<br>C -2.88854600 1.11972000 -0.00881500<br>C -2.45635400 2.43436800 -0.00136500<br>C -1.11645400 2.89502800 -0.00796000<br>C -0.69333800 4.25585100 0.00837200<br>C 0.69335700 4.25584800 0.00837200<br>C 1.11646700 2.89502300 -0.00796000<br>C 2.45636500 2.43435700 -0.00136500<br>C 2.88855100 1.11970700 -0.00881500<br>C 4.27268400 0.68047900 0.00874600<br>C 4.27267600 -0.68050500 0.00872200<br>C 2.88853600 -1.11971500 -0.00880600<br>C 2.45634700 -2.43436200 -0.00136100<br>H 1.36053800 -5.10885700 0.01858300<br>H -1.36056200 -5.10885000 0.01858300<br>H -3.22654100 -3.20039100 0.01488700<br>H -5.12445200 -1.34932700 0.02008700<br>H -5.12445400 1.34934000 0.02013000<br>H -3.22652600 3.20041700 0.01495700<br>H -1.36053600 5.10884900 0.01860900<br>H 1.36056000 5.10884300 0.01860900<br>H 3.22654000 3.20040200 0.01495700<br>H 5.12446000 1.34931600 0.02013000<br>H 5.12444500 -1.34935100 0.02008700<br>H 3.22652700 -3.20040600 0.01488700<br>H 0.00000000 0.00013400 1.53002300<br>Ir 0.00000000 -0.00000100 -0.01277900
**[IrP]$^{2-}$**<br>Charge Spin Multiplicity<br>-2 2<br>N 1.76842400 1.01397000 -0.00000200<br>N 3.82871300 3.06876300 0.00000000<br>N 5.88902300 1.01395500 -0.00000200<br>N 3.82873300 -1.04083800 -0.00000200<br>C 0.94361500 -0.10417700 -0.00000100<br>C -0.43929100 0.33274200 0.00000200	

C -0.43928700 1.69514000 -0.00000200<br>C 0.94364500 2.13205500 -0.00000100<br>C 1.37386200 3.44766600 0.00000000<br>C 2.71227500 3.90181100 0.00000000<br>C 3.13565700 5.26343100 0.00000100<br>C 4.52182300 5.26343300 0.00000300<br>C 4.94520200 3.90183400 0.00000000<br>C 6.28357900 3.44766800 -0.00000100<br>C 6.71382900 2.13209900 -0.00000100<br>C 8.09673700 1.69518500 -0.00000100<br>C 8.09673500 0.33278700 0.00000100<br>C 6.71380600 -0.10413300 -0.00000100<br>C 6.28358600 -1.41974100 -0.00000100<br>C 4.94517100 -1.87388600 -0.00000100<br>C 4.52179000 -3.23550500 0.00000100<br>C 3.13562300 -3.23550800 0.00000200<br>C 2.71224500 -1.87391000 -0.00000100<br>C 1.37386700 -1.41974300 -0.00000100<br>H -1.29164300 -0.33630400 0.00000500<br>H -1.29163400 2.36419200 -0.00000300<br>H 0.60371600 4.21502300 0.00000000<br>H 2.46757300 6.11655500 0.00000100<br>H 5.18990400 6.11655900 0.00000500<br>H 7.05372300 4.21503200 0.00000000<br>H 8.94908900 2.36423100 -0.00000200<br>H 8.94908500 -0.33626200 0.00000300<br>H 7.05373000 -2.18710000 0.00000000<br>H 5.18987000 -4.08863200 0.00000200<br>H 2.46754600 -4.08863700 0.00000300<br>H 0.60372500 -2.18710900 0.00000000<br>Ir 3.82872300 1.01396200 -0.00000700	

Reference

1. Connelly, S.J.; Wiedner, E.S.; Appel, A.M. Predicting the reactivity of hydride donors in water: Thermodynamic constants for hydrogen. *Dalton Trans.* **2015**, *44*, 5933–5938.
